# Single-cell transcriptomic analysis reveals CD8 + T cell heterogeneity and identifies a prognostic signature in cervical cancer

**DOI:** 10.1186/s12885-025-13901-x

**Published:** 2025-03-18

**Authors:** Rongbin Zhou, Yuli Xie, Zuheng Wang, Zige Liu, Wenhao Lu, Xiao Li, Chunmeng Wei, Xing Li, Fubo Wang

**Affiliations:** 1 Collaborative Innovation Centre of Regenerative Medicine and Medical BioResource Development and Application Co-constructed by the Province and Ministry, Guangxi Medical University, Nanning, Guangxi 530021 China; 2 Center for Genomic and Personalized Medicine, Guangxi key Laboratory for Genomic and Personalized Medicine, Guangxi Collaborative Innovation Center for Genomic and Personalized Medicine, Guangxi Medical University, Nanning, Guangxi 530021 China; 3 Department of Urology, The First Affiliated Hospital of Guangxi Medical University, Guangxi Medical University, Guangxi, 530021 China; 4 School of Life Sciences, Guangxi Medical University, Nanning, Guangxi 530021 China; 5 Department of Urology, Affiliated Tumor Hospital of Guangxi Medical University, Guangxi Medical University, Nanning, Guangxi 530021 China; 6 School of Public Health, Guangxi Medical University, Nanning, Guangxi 530021 China; 7 Department of Obstetrics and Gynecology, Shanghai General Hospital, School of Medicine, Shanghai Jiao Tong University, No. 85, Wujin Road, Hongkou District, Shanghai, 200080 China; 8 No. 22, Shuangyong Road, Qingxiu District, Nanning City, Guangxi Zhuang Autonomous Region 530021 China

**Keywords:** Cervical cancer, CD8 + T cell, Machine learning, Single-cell RNA-seq, Molecular docking

## Abstract

**Background:**

In recent years, immunotherapy has made significant progress. However, the understanding of the heterogeneity and function of T cells, particularly CD8 + T cells, in cervical cancer (CESC) microenvironment remains insufficient. We aim to characterize the heterogeneity, developmental trajectory, regulatory network, and intercellular communication of CD8 + T cells in cervical squamous cell carcinoma and to construct a prognostic risk model based on the transcriptomic characteristics of CD8 + T cells.

**Methods:**

We integrated single-cell RNA sequencing data from CESC tumor samples with bulk transcriptome data from TCGA and GEO databases. We identified CD8 + T cell subsets in the CESC microenvironment, revealing significant interactions between CD8 + T cells and other cell types through intercellular communication analysis. Pseudotime trajectory analysis revealed dynamic transcriptional regulation during CD8 + T cell differentiation and functional acquisition processes. We constructed a transcriptional regulatory network for CESC CD8 + T cells, identifying key transcription factors. Based on CD8 + T cell-related genes, a prognostic risk model comprising eight core genes was developed and validated using machine learning.

**Results:**

We identified four distinct CD8 + T cell subsets, namely progenitor, intermediate, proliferative, and terminally differentiated, each exhibiting unique transcriptomic characteristics and functional properties. CD8 + T cell subsets interact with macrophages through different ligand-receptor networks, including the CCL-CCR signaling pathway and costimulatory molecules. Sorafenib was identified as a potential immunotherapeutic drug through drug screening. Experimental validation demonstrated that sorafenib enhances the cytotoxicity of CD8 + T cells by increasing the secretion of IFN-γ and TNF-α, thereby significantly inhibiting the invasiveness and survival of CESC cells.

**Conclusions:**

Our study provides valuable insights into the heterogeneity and functional diversity of CD8 + T cells in CESC. We demonstrate that a CD8 + T cell-related prognostic signature may serve as a potential tool for risk stratification in patients with CESC. Additionally, our finding suggests that sorafenib could be a promising therapeutic candidate for improving antitumor immunity in this patient population.

**Supplementary Information:**

The online version contains supplementary material available at 10.1186/s12885-025-13901-x.

## Background

Cervical cancer (CESC) remains one of the leading malignancies threatening women’s health worldwide [1]. Despite advances in treatment modalities, the prognosis for CESC patients remains unsatisfactory [2]. Growing evidence indicates that the tumor microenvironment (TME) is crucial in cancer progression and therapeutic responses [3]. Immune cells within the TME, particularly CD8 + T cells, are key players in anti-tumor immunity [4]. CD8 + T cells are the primary effector cells of cellular immunity, exerting anti-tumor effects through direct cytotoxicity against tumor cells and the secretion of cytokines [5]. Thus, understanding the characteristics and functions of immune cells, especially CD8 + T cells, in the CESC microenvironment is crucial for developing effective immunotherapeutic strategies [6].

Recent studies have revealed the heterogeneity of immune cells within the TME, underscoring the need for more precise characterization of immune cell subpopulations [7, 8]. Tumor-infiltrating CD8 + T cells inhibit tumor growth in various malignancies and are associated with favorable patient prognosis [9, 10]. However, the heterogeneity of T cells in CESC and their roles in shaping the TME remain largely unexplored. Conventional bulk RNA sequencing (RNA-seq) profiles gene expression in tumor samples but fails to capture single-cell heterogeneity [11]. Single-cell RNA sequencing (scRNA-seq) has revolutionized our understanding of cellular heterogeneity and has been successfully applied to various cancers [12]. However, systematic studies integrating scRNA-seq and bulk RNA-seq data to delineate the T cell landscape in CESC are lacking, hindering the development of precision immunotherapy.

Developing prognostic models based on CD8 + T cell molecular characteristics holds great potential for risk stratification and personalized treatment planning [13, 14]. Current methods, such as immune cell infiltration scores and immune subtype classifications, are widely used to predict clinical outcomes in various cancers [15]. However, these methods primarily assess immune cells at the overall level, failing to focus on the molecular states of specific cell subtypes and their prognostic impact.

Identifying potential therapeutic targets and drug candidates is crucial to improving the treatment of CESC [16]. Sorafenib, a multi-kinase inhibitor, has shown promising results in treating various malignancies [17, 18]. However, its potential in CESC immunotherapy remains unexplored.

This study aims to comprehensively analyze the heterogeneity and functional dynamics of CD8 + T cells in the TME of CESC by integrating scRNA-seq and bulk RNA-seq data. We aim to identify key CD8 + T cell subpopulations, delineate their developmental trajectories, and uncover regulatory networks and intercellular communications shaping the TME. Additionally, we aim to construct a prognostic model based on CD8 + T cell molecular characteristics and identify potential therapeutic targets and drug candidates. Our findings will expand the understanding of CD8 + T cell mechanisms in CESC, provide new insights into TME characteristics, and showcase the immense potential of single-cell omics in precision oncology, ultimately facilitating the development of effective immunotherapeutic strategies for CESC patients.

## Methods

### Data sources

scRNA-seq data from four purified CESC tumor samples in GSE171894 were downloaded from the Gene Expression Omnibus (GEO) database (http://www.ncbi.nlm.nih.gov/geo/*).* Additionally, bulk RNA-seq data and clinical information for 309 CESC patients were obtained from the Cancer Genome Atlas (TCGA) database (https://portal.gdc.cancer.gov*).* After excluding samples without survival data, 293 patients were included for further analysis. Moreover, transcriptomic and matched clinical data for patients in the IMvigor210 cohort who received anti-PD-L1 therapy were collected (http://research-pub.gene.com/IMvigor210CoreBiologies*).* To validate the prognostic ability of the model, GSE44001 (*n* = 300) was selected as an external validation dataset. Data from GEO were integrated and batch effects were corrected using the “ComBat” algorithm in the “sva” package [19]. All bulk RNA sequencing data were log_2_ transformed for subsequent analysis.

### Initial processing workflow for scRNA-seq data

scRNA-seq data integration was performed using Seurat package (v4.3.0.1) in R (v4.2.1). Seurat objects were created after filtering for genes in > 3 cells with 200–6000 features. Cells with > 25% mitochondrial genes were excluded using ‘PercentageFeatureSet’. Gene expression was normalized using the ‘LogNormalize’ method [19].

### Cell type identification

Using the ‘FindAllMarkers’, we identified genes with significant expression in specific cell clusters, marking them as potential biological markers. This process was guided by the following criteria: a minimum percentage threshold of 0.25, a log fold change threshold also at 0.25, and a *P*-value of less than 0.05. Additionally, for acquiring standard markers used to distinguish different cell types, we consulted the Cell Markers database (http://xteam.xbio.top/CellMarker/index.jsp#*).*

### Intercellular communication

The CellChatDB.human database was used to annotate ligand-receptor interactions, focusing on secretory signaling pathways. Communication probabilities were calculated for overexpressed ligand-receptor pairs, with significance assessed via permutation tests. The resulting network was analyzed for the TopDC pathway and interaction strength, and visualized using bubble plots to display intercellular communication patterns [20].

### Pseudotime trajectory analysis

Cell trajectory analysis was performed using Monocle 2 (v2.26.0) following Seurat-based cell clustering. Genes with average expression ≥ 0.1 were selected for trajectory analysis. Data dimensionality was reduced using “DDRTree”, and cells were ordered using the “orderCells”. Key transition genes were identified through BEAM analysis and visualized via branched heatmaps. Gene expression patterns were plotted using jitter, violin, and pseudotime plots. The trajectory was displayed on a tSNE plot with dynamic expression heatmaps. Gene cluster functions were analyzed using clusterProfiler for GO enrichment [21].

### Transcription factor regulatory network

A transcription factor-target gene regulatory network was constructed using the pySCENIC workflow (version 0.12.1) [22]. First, gene co-expression analysis was performed using the GRNBoost2 algorithm from the “Arboreto” package to form a co-expression network. Next, RcisTarget was employed for cis-regulatory motif analysis to identify significantly enriched direct targets and construct regulatory network modules (regulons). Finally, AUCell was utilized to score the target genes of regulatory factors, calculate regulon activity, and classify cells based on this activity. The network was visualized using Cytoscape software (version 3.9.1), illustrating the regulatory relationships.

### Machine learning signature construction and validation

To construct and validate a machine learning (ML)-based signature, we adopted a comprehensive approach integrating 101 different combinations of 10 distinct ML algorithms to develop a prognostic signature with significant accuracy and stability. The ML algorithms used in this study included CoxBoost, Elastic Net (Enet), Survival Support Vector Machine (survival-SVM), Lasso, Partial Least Squares Regression for Cox (plsRcox), Ridge, Random Survival Forest (RSF), Stepwise Cox, Supervised Principal Components Analysis (SuperPC), and Generalized Boosted Regression Modeling (GBM) [23]. Notably, some ML algorithms, such as CoxBoost, Lasso, RSF, and Stepwise Cox, incorporate feature selection capabilities.

The specific ML process involved: (1) Using univariate Cox regression analysis to preliminarily identify CD8T cell high-variance genes (CD8TGs) with prognostic potential in the TCGA cohort; (2) Fitting predictive models on the TCGA dataset using 101 algorithm combinations; (3) Further validating model performance in the external validation cohort GSE44001; and (4) Calculating the C-index for all cohorts to identify the model with the highest average C-index as the best model. Additionally, we developed a CD8T-related risk score (TRS) for CESC.

Using the best model, patients were classified into high-risk and low-risk groups based on the optimal threshold derived from the TCGA cohort and the independent validation cohort. The prognostic value and predictive accuracy of the best model were evaluated using Receiver Operating Characteristic (ROC) curves and Kaplan-Meier curves.

### Immune cell infiltration analysis

The ESTIMATE algorithm was utilized to calculate TME scores, including the immune score, stromal score, and ESTIMATE score. The abundance of various immune cells was estimated using the CIBERSORT method [24]. RNA sequencing data and clinical parameters of urothelial carcinoma patients from the IMvigor210 immunotherapy cohort were used to categorize patients into responder and non-responder subgroups [25]. The Wilcoxon test was then employed to compare the differences in risk scores between the two groups. Additionally, we analyzed the expression correlation of immune checkpoint molecules between the two risk clusters and the abundance differences of various immune cells in low-risk and high-risk CESC, which were displayed using box plots. To explore potential mechanisms in the high-risk and low-risk groups, GSEA analysis was performed using the c2kegg gene set [26], with thresholds set for a normalized enrichment score (NES) greater than 1 and a nominal p-value less than 0.05.

### Molecular docking using pyrx software

Approximately 2,162 FDA-approved drugs were retrieved from the DrugBank database and analyzed for structure-based repositioning. First, the three-dimensional structures of core CD8T targets in CESC were downloaded from the PDB and AlphaFold Protein Structure databases. The two-dimensional structures of the molecular ligands were converted to three-dimensional structures using PyMOL software. The protein molecules were then dehydrated, hydrogenated, and charged using AutoDock tools. Subsequently, virtual drug screening was performed using PyRx software, docking the preprocessed protein molecules with the 2,162 FDA-approved drug molecules [27]. Small molecules with a binding free energy of less than − 6 kcal/mol were selected as potential targeted drugs. The docking results were sorted and evaluated using AutoDock’s analysis functions, ultimately displaying the small molecule structures with the best binding affinities to the protein. To eliminate global structural bias and non-convergent conformations, structures with RMSD/ub ≥ 5.0 Å and RMSD/lb ≥ 5.0 Å were excluded. Considering docking evaluation metrics such as binding affinity, RMSD/ub, and RMSD/lb, along with the ranking and application of the drug in cancer immunotherapy [28, 29], sorafenib was ultimately selected as a potential immunotherapy candidate for cervical squamous cell carcinoma. The molecular docking results were visualized using PyMOL.

### Cell culture

Human CESC cells (HeLa, STCC10603) were obtained from Servicebio (Wuhan, China), authenticated by short tandem repeat profiling, and tested for mycoplasma contamination. HeLa cells were selected as a representative model for CESC due to their widespread use in CESC research and strong immunogenic characteristics [30]. HeLa cells were cultured in DMEM-H medium (GUMD-B304, HyCyte), supplemented with 10% fetal bovine serum (FBS-C550, HyCyte) and 1% penicillin-streptomycin (G4003-100ML, HyCyte), in a humidified atmosphere at 37 °C with 5% CO₂. Human CD8 + T cells were provided by Shanghai Zhongqiao Xinzhou Biotechnology Co., Ltd. They were cultured in RPMI-1640 medium containing 10% fetal bovine serum and 1% penicillin-streptomycin, at 37 °C in a humidified incubator with 5% CO₂. Prior to treatment, CD8 + T cells were activated with anti-CD3/CD28 antibodies for 48 h.

### Enzyme-linked immunosorbent assay

The levels of IFN-γ and TNF-α were measured using a commercial ELISA kit (Servicebio, GEH0006, 88-7346) according to the manufacturer’s instructions. CD8 + T cells were co-cultured with varying concentrations of sorafenib (HY-10201, MCE) in a 6-well plate. Based on the immunomodulatory properties reported in previous literature and the safety recommendations provided by the manufacturer [31, 32], sorafenib concentrations of 1, 1.4, 3, and 5 µM were selected to establish a dose-response relationship in vitro. The culture supernatant was collected and centrifuged at 3,000 rpm for 15 min at 4 °C. Standard solutions and samples (100 µL) were added to pre-coated wells and incubated at 37 °C for 1–2 h. After washing, 100 µL of biotinylated antibody was added and incubated at 37 °C for 1 h. Subsequently, 100 µL of enzyme conjugate was added and incubated at 37 °C for 30 min. The colorimetric reaction was performed using 100 µL of TMB substrate, incubating at 37 °C in the dark for 10–30 min, until a clear color gradient appeared in the diluted standard wells. The reaction was then terminated with H₂SO₄ (2 M). The optical density was measured at 450 nm using an enzyme-linked immunosorbent assay reader.

### Flow cytometry assay

Flow cytometry was performed to measure the production of IFN-γ and TNF-α. CD8 + T cells were divided into two groups: the control group and the sorafenib treatment group. Sorafenib (3 µM) was added to the treatment group, and the cells were incubated at 37 °C in a humidified atmosphere with 5% CO₂ for 24 h. Untreated CD8 + T cells served as the control. A total of 1 × 10^6 cells were collected and centrifuged at 1200 rpm for 5 min. The cell pellet was resuspended in PBS containing 1‰ Zombie Yellow (Biolegend) and incubated at room temperature in the dark for 20 min. After washing with PBS containing 1% BSA, cells were incubated for 10 min at 4 °C in the dark with 1 µL Fc receptor blocking solution in 50 µL PBS (1% BSA). After incubating the cells in the dark at 4 °C for 10 min, they were fixed with 4% paraformaldehyde at room temperature for 15 min. The cells were then washed twice with PBS to remove the fixative. Subsequently, the cells were resuspended in 100 µL permeabilization buffer (Saponin, ED-8648) and incubated at room temperature in the dark for 20 min. Next, 5 µL of human IFN-γ antibody (Biolegend, 383303) and 5 µL of human TNF-α-PE antibody (Biolegend, 502908) were added. Following incubation, cells were washed by centrifugation with 2 mL flow cytometry staining buffer at 300 × g for 5 min. The supernatant was discarded, and the cells were resuspended in 500 µL of flow cytometry staining buffer for analysis. Flow cytometry data were acquired using a BD FACSAri flow cytometer and analyzed with FlowJo v10.8.1. The mean fluorescence intensity (MFI) of IFN-γ and TNF-α in the CD8 + T cell population was measured.

### Invasion assay

To investigate whether sorafenib-treated CD8 + T cells affect the invasiveness of HeLa cells, a Matrigel invasion assay was conducted. CD8 + T cells were co-cultured with 3 µM sorafenib for 48 h, with untreated CD8 + T cells serving as the control. Matrigel (Corning, 356234) was diluted at a 1:8 ratio in serum-free medium, and 60 µL was added to the upper chamber of Transwell inserts (Corning, 3422) to simulate the basement membrane. After incubation at 37 °C for 4 h, 1 × 10^5 HeLa cells were seeded into the upper chamber, while the lower chamber contained 600 µL of CD8 + T cell supernatant as a chemoattractant. After 48 h, cells that had invaded the lower surface were fixed with paraformaldehyde for 20 min, stained with 0.1% crystal violet for 15 min, and then randomly selected five fields under a microscope for counting.

### Co-culture of CD8 + T cells and HeLa cells

CD8 + T cells were divided into two groups: a control group and a sorafenib treatment group. Sorafenib (3 µM) was added to the treatment group, which was incubated at 37 °C in a humidified atmosphere with 5% CO₂ for 24 h. Untreated CD8 + T cells served as the control. According to previous tumor immunotherapy models [33], sorafenib-treated and untreated CD8 + T cells were co-cultured with HeLa cells in a 96-well plate at a 5:1 ratio (6,000 cells per well). HeLa cells cultured alone served as the blank control. Co-cultures were maintained under standard conditions (37 °C, 5% CO₂) for 72 h. After co-culture, PBS was added to remove the suspended CD8 + T cells. HeLa cell proliferation was assessed using a CCK-8 assay (GUTK-R00, HyCyte). 100 µL of fresh medium containing 10 µL of CCK-8 reagent was added to each well, followed by incubation for 1 h. The optical density was measured at 450 nm using a microplate reader. Proliferation rates of HeLa cells were compared between groups, with a decrease in optical density indicating reduced proliferation.

### Statistical analysis

In our study, we utilized the Wilcoxon rank-sum test, a non-parametric method, to identify differences between two sample groups and employed Spearman rank correlation analysis to assess the monotonic relationships between variables. The log-rank test was used to compare survival curves between groups. For survival analysis and the evaluation of the diagnostic capability of relevant prognostic genes, we used Kaplan-Meier survival curves and the area under the ROC curve (AUC). All statistical analyses and computations were performed using R software (version 4.2.1), Python (version 3.8.18), and GraphPad (version 10.1.2). All experiments were repeated at least three times. A p-value of less than 0.05 was considered statistically significant. These methods provide a comprehensive and rigorous framework for accurately assessing the association between various biomarkers and patient prognosis.

## Results

### Cell composition of CESC

**Fig. 1:**
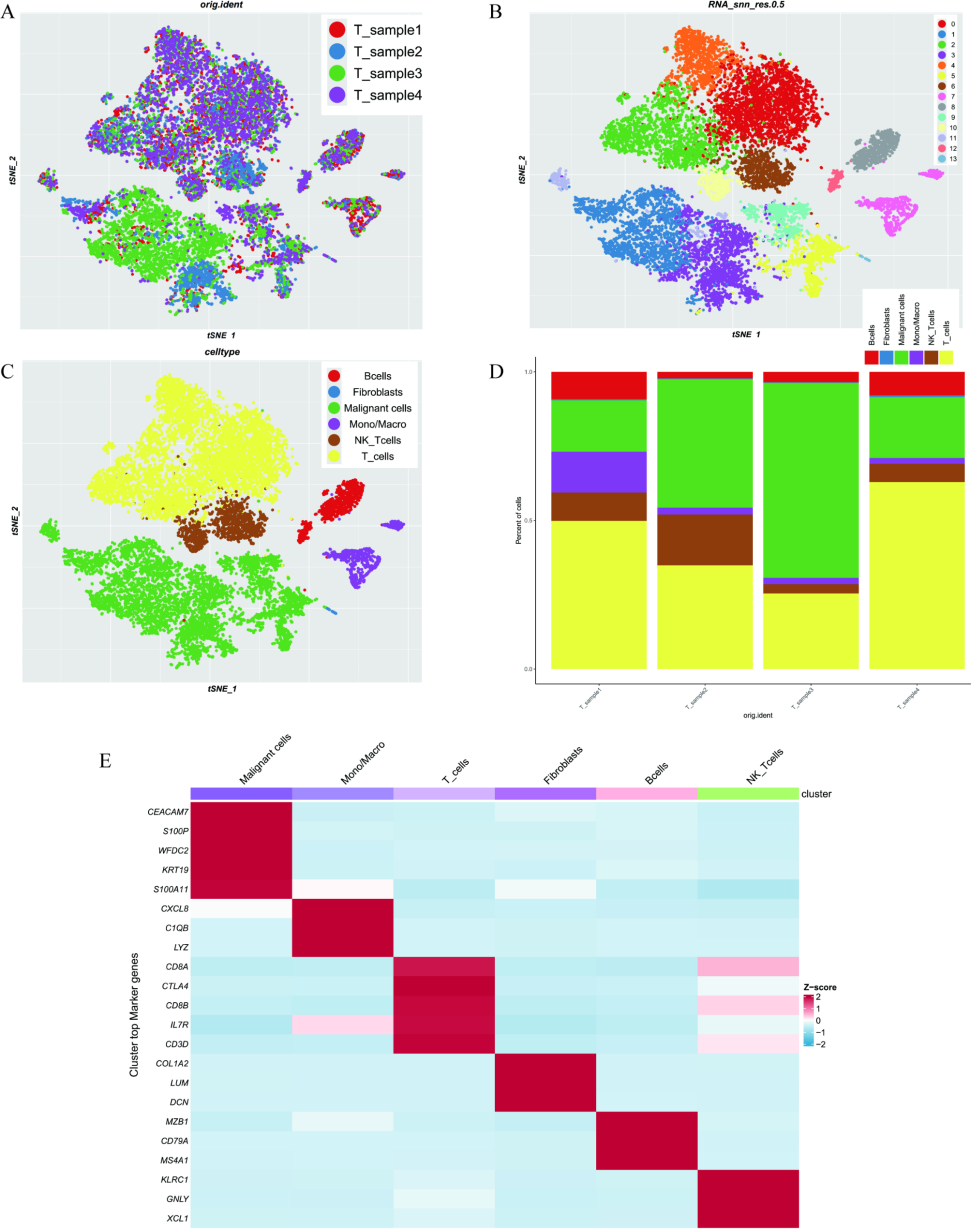
Single-cell transcriptome data from four CESC patients, including the distribution of different cell populations, cell type annotations, and their characteristic gene expression patterns. (**A**) Cellular composition of CESC, derived from four patients. (**B**) Cells divided into 14 distinct clusters. (**C**) Cells further classified into six major types. (**D**) Proportion of T cells across all samples. (**E**)Gene expression markers of different cell types. CESC, cervical cancer

To investigate the cellular composition and molecular characteristics of CESC, single-cell RNA sequencing data from four CESC samples were collected. After quality control, a total of 21,676 cells were obtained, with 4,968, 4,669, 7,457, and 4,582 cells in each sample, respectively (Fig. 1A). During data analysis, cells were classified into 14 distinct clusters (Fig. 1B), which were further categorized into six primary cell types (Fig. 1C). T cells constituted over 25% of the cells in all samples (Fig. 1D). Malignant cells (8,706 cells; clusters 1, 3, 5, 9, and 11) prominently expressed epithelial markers, including CEACAM7, S100P, KRT19, S100A11, and WFDC2. B cells (1,185 cells; clusters 8 and 12) exhibited significant expression of CD79A, MZB1, and MS4A1. T cells (8,882 cells; clusters 0, 4, 2, and 10) were characterized by the expression of CD8A, GZMH, CTLA4, and CD8B. Macrophages (1,039 cells; cluster 7) were identified by markers including CXCL8, C1QB, and LYZ. Cancer-associated fibroblasts (78 cells; cluster 13) specifically expressed COL1A2, DCN, and LUM. Other clusters displayed characteristics of natural killer cells, marked by KLRC1, GNLY (1,786 cells; Fig. 1E). All marker genes in the single-cell data are listed in Supplementary Table S1.

### Distribution and functional heterogeneity of CD8 + T subsets in CESC

**Fig. 2:**
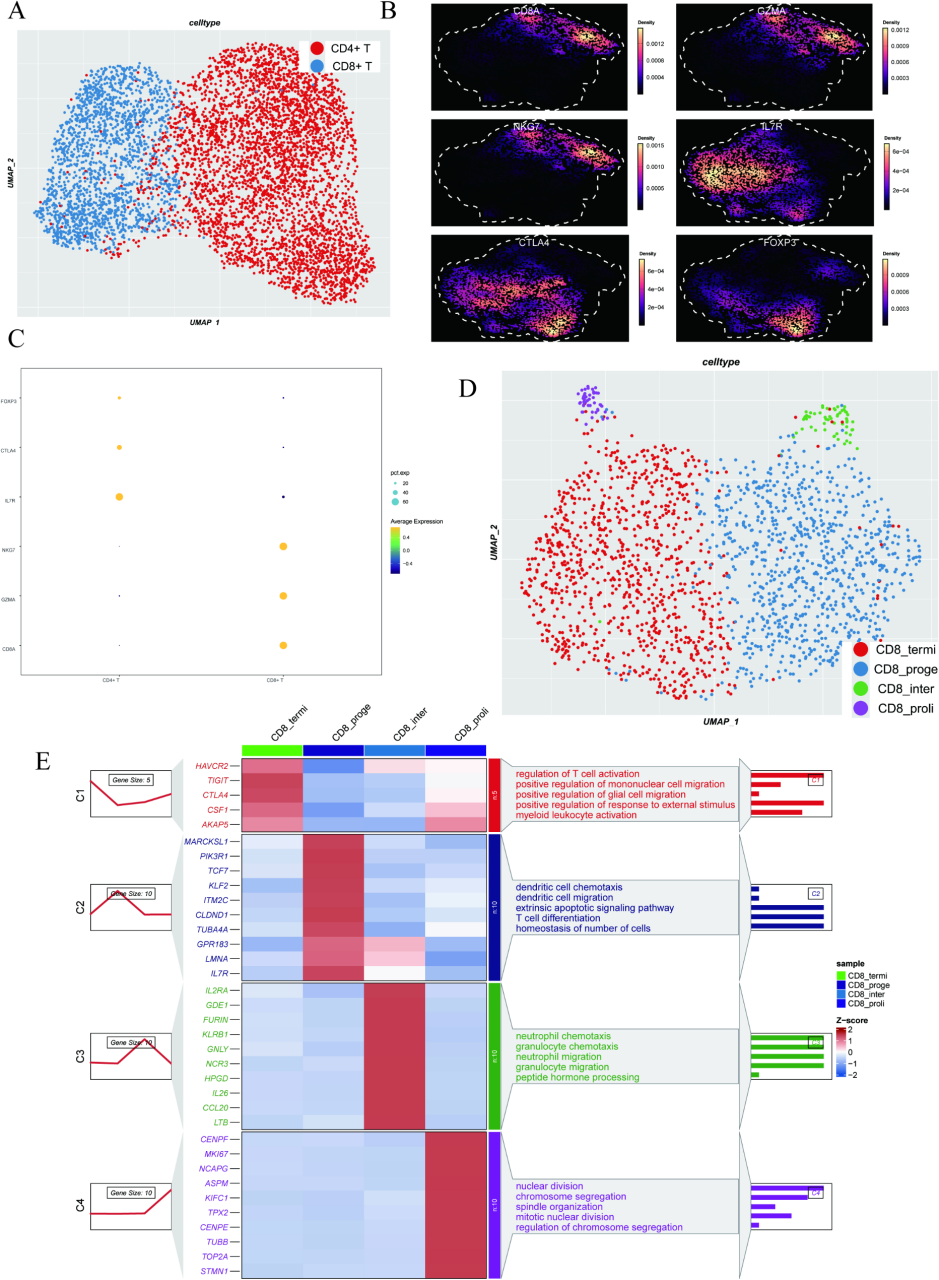
Identification and characterization of CD8 + T cell subpopulations. (**A**) t-SNE plot of T cells annotated as CD4+ (red) and CD8+ (blue) T cells. (**B**) Expression density of marker genes for CD4 + and CD8 + T cells in the t-SNE space. (**C**) Bubble plot showing the percentage of cells expressing each gene (size) and the average expression level (color) in CD4 + and CD8 + T cells. (**D**) t-SNE plot revealing four CD8 + T cell subpopulations: CD8_termi (red), CD8_prolif (purple), CD8_inter (blue), and CD8_proge (green). (**E**) Heatmap displaying the expression dynamics and functional enrichment of marker genes across CD8 + T cell subpopulations. Left: expression trends (C1–C4). Middle: Z-scored expression levels of representative marker genes. Right: most enriched GO terms for each subpopulation

Figure 2A illustrates the further classification of T cells into CD4 + T cells (6,040 cells, red) and CD8 + T cells (2,842 cells, blue), providing a foundation for exploring the heterogeneity of CD8 + T cells in the disease. Figure 2B shows the expression density of marker genes in the t-SNE space, with CD8A, GZMA, and NKG7 highly expressed in the upper right region, while IL7R, CTLA4, and FOXP3 partially overlap in the central to lower regions. The bubble chart in Fig. 2C indicates that FOXP3, CTLA4, and IL7R are preferentially expressed in CD4 + T cells, whereas CD8A, GZMA, and NKG7 are highly expressed in CD8 + T cells. Figure 2D demonstrates the further subdivision of CD8 + T cells into four subsets: terminally differentiated cells (1,408 cells, CD8_termi), proliferative cells (68 cells, CD8_prolif), intermediate differentiated cells (90 cells, CD8_inter), and progenitor cells (1,276 cells, CD8_proge), highlighting intra-cluster heterogeneity. The heatmap in Fig. 2E depicts the expression and functional enrichment of marker genes in different subsets [34], revealing the functional characteristics of these subsets in biological processes such as T cell activation, mitosis, and T cell differentiation.

### Ligand-receptor interactions between CD8⁺ T cell subsets and the tumor immune microenvironment

**Fig. 3:**
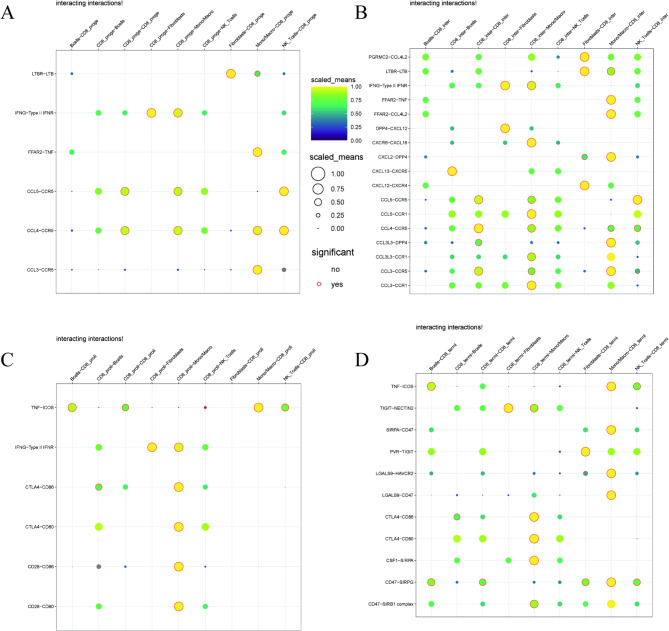
Ligand-receptor interactions between CD8 + T cell subsets and immune cells in the tumor microenvironment. The size of the dots represents the molecular expression level, the color intensity indicates the interaction strength, and the red outlines denote significant interactions. (**A**) Dot plot highlighting significant ligand-receptor interactions between CD8_proge cells and various immune cell types. (**B**) Dot plot showing the potential chemokine-mediated recruitment of macrophages by CD8_inter cells. (**C**) Dot plot revealing the communication patterns between CD8_prolif cells and monocytes/macrophages. (**D**) Dot plot identifying CD8_termi cells as receptors for macrophage-derived ligands

**Fig. 4:**
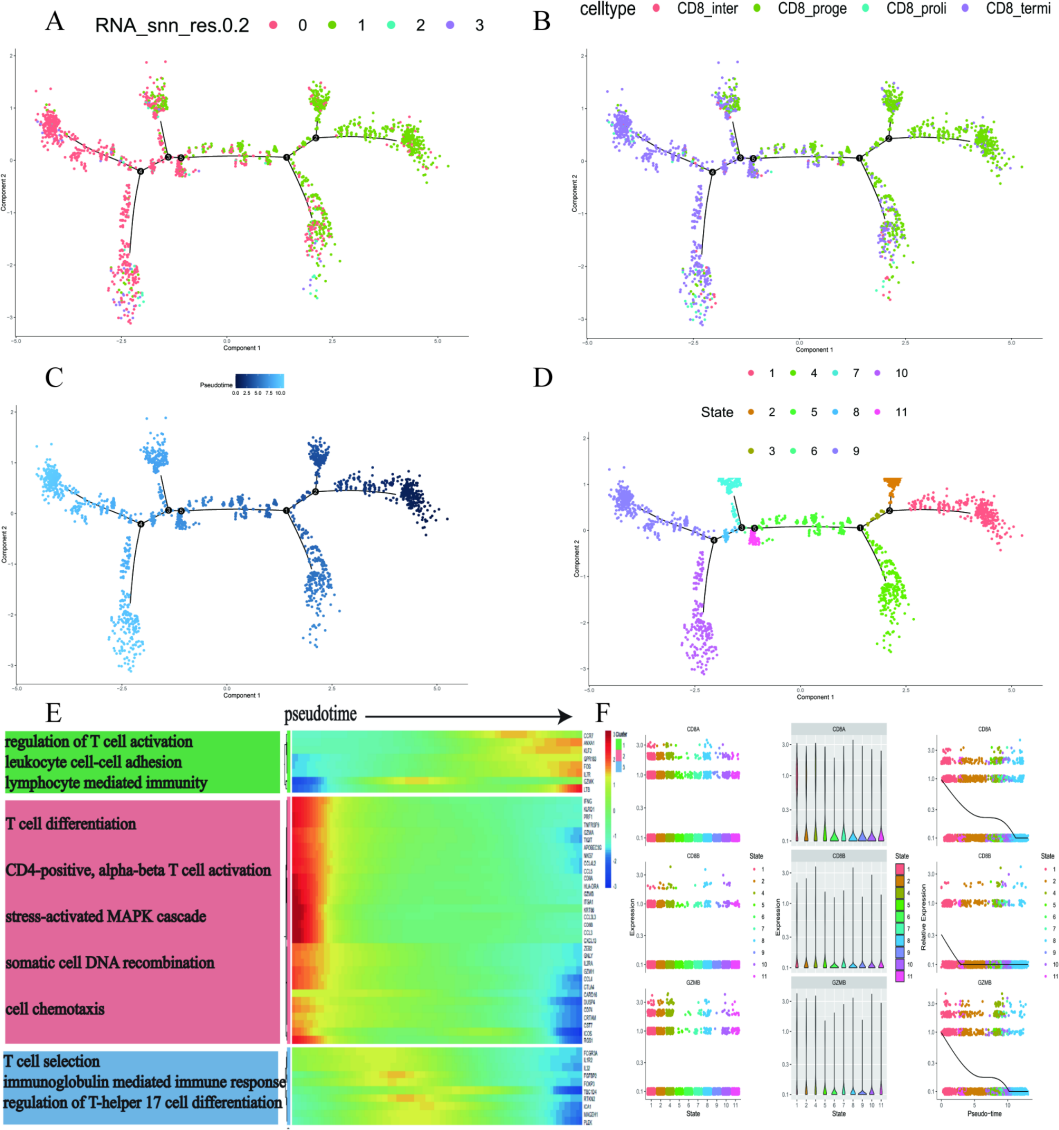
Pseudotime trajectory analysis reveals developmental paths and transcriptomic dynamics of CD8⁺ T cell subsets. (**A**-**D**) Pseudotime trajectory of CD8⁺ T cells, colored by (**A**) CD8 + T cell subclusters, (**B**) celltype, (**C**) Pseudotime, and (**D**) State. (**E**) Three gene clusters with distinct dynamic expression patterns identified by differential expression analysis between CD8⁺ T cell subsets at different pseudotimes. (**F**) Gene regulatory dynamics along the root-to-cell fate trajectory

By analyzing the potential interactions between CD8⁺ T cell subsets and other immune cells in the TME, we identified distinct communication patterns exhibited by CD8⁺ T cells at different differentiation stages compared to macrophages/monocytes. Figure 3A shows significant interactions between CD8_proge cells and macrophages, including LTBR-LTB, FGFR2-TNF, and several CCL-CCR pairs, suggesting paracrine signaling pathways that regulate the immune microenvironment (Supplementary Table S2A-B). Figure 3B highlights numerous potential interactions between CD8_inter cells and macrophages, such as CCL4-CCR5 and CXCL9-DPP4, indicating their role in chemokine-mediated macrophage recruitment (Supplementary Table S2C-D). Figure 3C reveals a communication network dominated by proliferative CD8 + T cells interacting with monocytes/macrophages through co-stimulatory pathways, including CTLA4-CD86 and CD28-CD80, suggesting the formation of active immunological synapses. Finally Fig. 4D (Supplementary Table S2E-F) highlights terminally differentiated CD8_termi cells as mediators of macrophage-derived ligand-receptor interactions, with key pathways involving TNF-ICOS and TIGIT-NECTIN2, emphasizing their extensive molecular communication network with macrophages/monocytes in the TME (Supplementary Table S2G-H). These findings underscore the complex and organized intercellular communication network formed by CD8⁺ T cell subsets with macrophages/monocytes in the tumor immune microenvironment through mechanisms involving cytokines, chemokines, and co-stimulatory molecules.

### Pseudotime analysis reveals developmental trajectories and transcriptomic dynamics of CD8⁺ T cell subsets

Pseudotime trajectory analysis revealed the dynamic transcriptional changes and developmental pathways of CD8⁺ T cell subsets (Fig. 4A-D). All CD8⁺ T cells were mapped onto a continuous differentiation trajectory with a starting point (root) and six endpoints, presenting eight distinct differentiation states (State 1–9). CD8_proge cells were located at the initial stage of differentiation (State 1), while mature CD8_termi (State 8–11), proliferative cells (CD8_prolif, State 10), and intermediate differentiated cells (CD8_inter, State 7, 10) emerged over pseudotime. This analysis highlights the continuity of CD8⁺ T cell differentiation and provides deeper insights into its developmental regulatory mechanisms. Differential expression analysis identified three gene clusters: Cluster 1 genes were upregulated over pseudotime and enriched in T cell activation pathways, while Cluster 2 genes were downregulated during differentiation and significantly enriched in pathways related to T cell differentiation and chemotaxis (Fig. 4E). Furthermore, trajectory analysis revealed the dynamic decrease in the expression of the CD8⁺ T marker CD8A (Fig. 4F), indicating that transcriptional changes play a crucial role in activation, differentiation, and immune responses during CD8⁺ T cell development.

### Transcriptional regulatory networks and functional analysis of core factors in CD8 + T cell subsets

**Fig. 5:**
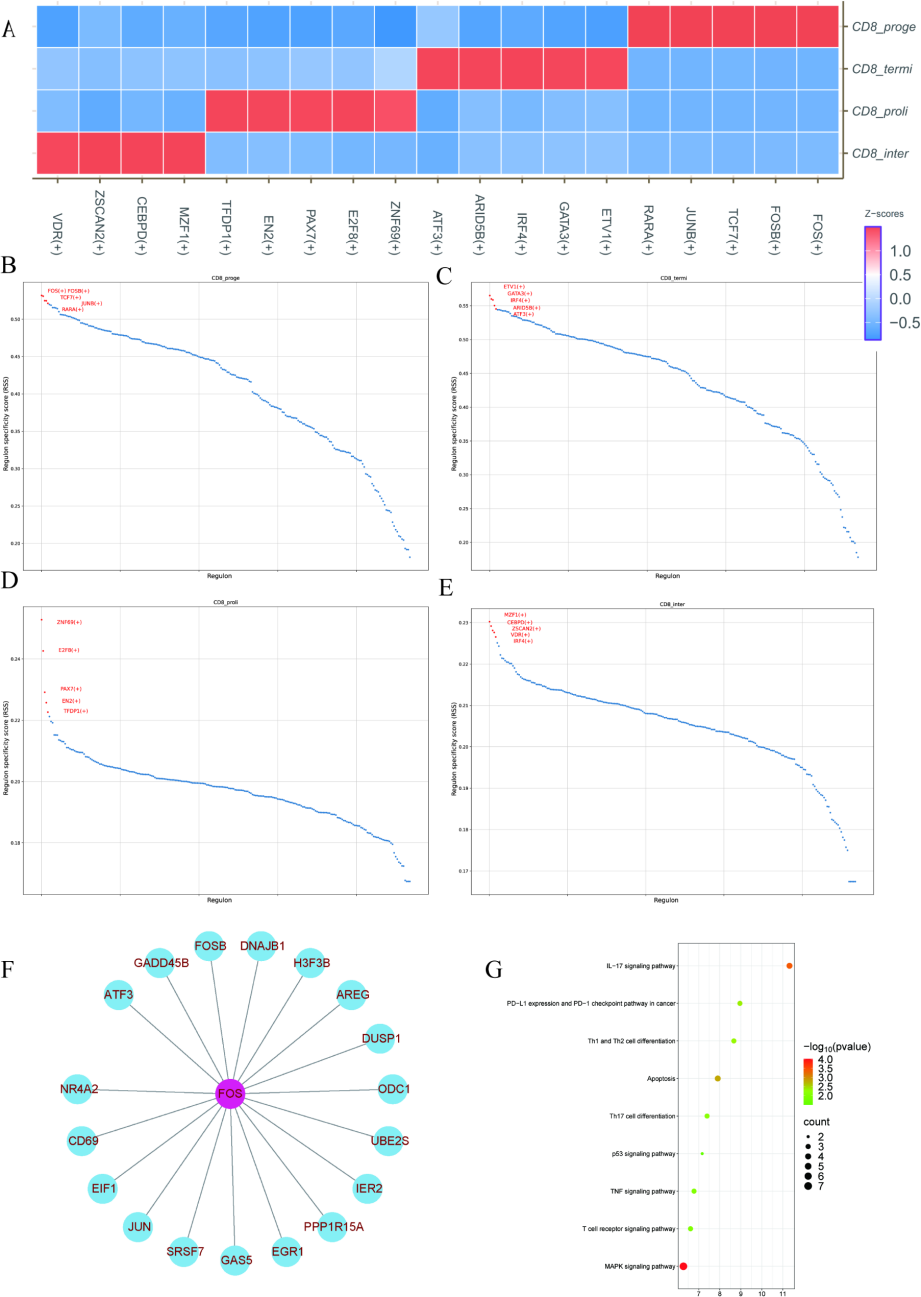
Transcriptional regulatory networks and core factors in CD8 + T cell subsets. (**A**) Heatmap of transcription factor activity across CD8 + T cell subsets. (**B**-**E**) RSS plots showing the top 5 TFs in each CD8 + T cell subset. (**F**) Network diagram illustrating the central regulatory role of FOS in CD8_proge and its top 20 downstream target genes. (**G**) Functional enrichment analysis of FOS downstream target genes

By analyzing the transcription factor regulatory networks of the four CD8 + T cell subsets, distinct transcription factor activity patterns were identified for each subset. The heatmap illustrates the transcription factor (TF) regulatory networks in the four CD8 + T cell subsets, showing enhanced activity of factors such as TCF7, FOS, and RARA in CD8_proge cells, while ETV1, GATA3, and IRF4 are more active in CD8_termi cells (Fig. 5A). The RSS score plots further highlight the five most important regulatory factors in each subset (Fig. 5B-E). Notably, the network diagram positions FOS as the central regulatory TF in CD8_proge cells, interacting with downstream target genes such as FOSB, GADD45B, CD69, NR4A2, and EGR1 (Fig. 5F). This network emphasizes the critical role of FOS in coordinating gene expression programs essential for the early differentiation and functional specification of progenitor CD8 + T cells. Additionally, FOS is identified as a key central regulatory factor in progenitor cells, with its downstream target genes involved in multiple immune-related pathways, including the MAPK signaling pathway, p53 signaling pathway, Th1/Th2 differentiation, and TNF signaling pathway. These findings reveal the pivotal role of FOS in the early differentiation and immune functional specialization of CD8 + T cells (Fig. 5G).

### Establishment and validation the predictive model

**Fig. 6:**
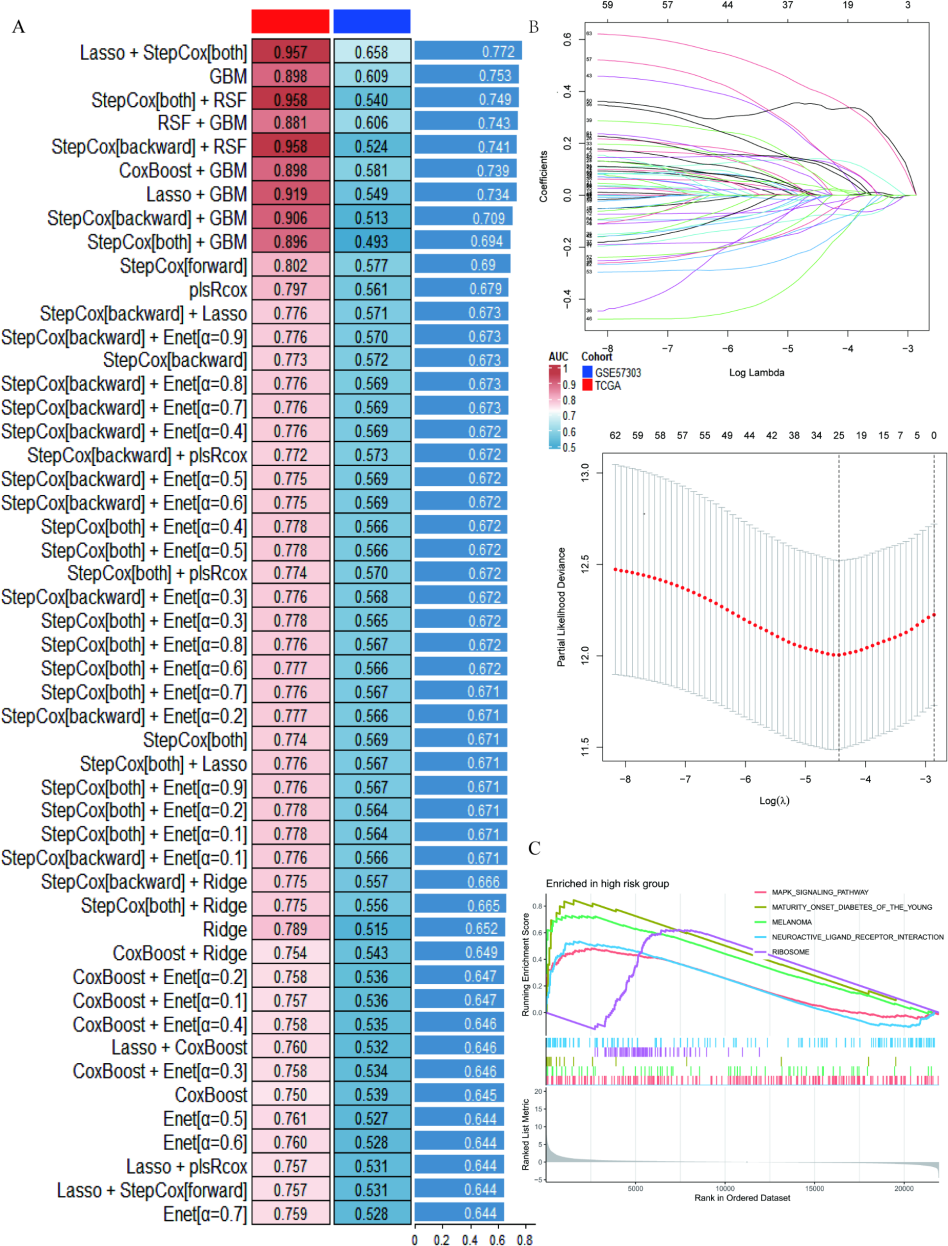
Development and characteristics of the CD8 + T cell-related prognostic signature. (**A**) Distribution of C-index for Lasso and StepCox combined models in the TCGA dataset (showing only the top 50 C-index rankings). (**B**) The process of determining the optimal λ value and coefficient selection in Lasso regression. (**C**) Biological processes and KEGG pathways enriched in the high-risk group

In the preliminary analysis, 320 differentially expressed genes associated with CD8 + T cells were identified. To explore the potential prognostic value of these CD8 + T cell-related genes, a univariate Cox proportional hazards regression analysis was conducted on the TCGA CESC training cohort. This analysis revealed that 64 CD8 + T cell-associated genes were significantly correlated with overall survival. Based on the univariate Cox analysis, these 64 significant genes were further evaluated to construct a CD8 + T cell-based prognostic signature (Supplementary Table S3). These genes were input into an integrated ML program to develop the TRS. We evaluated the predictive performance of 101 different ML algorithm combinations on the TCGA and GSE57303 datasets. The combination of Lasso and StepCox (used simultaneously) achieved the highest average C-index of 0.802 on the TCGA dataset, making it the optimal model (Fig. 6A; Supplementary Table S4). Figure 6B shows the process of selecting Lasso coefficients and λ, indicating that the optimal λ value was determined through Lasso regression, with the partial likelihood deviation reaching its minimum at λ = 0.0673. The TRS developed based on Lasso regression analysis includes 8 genes and calculates the TRS score (risk score) for CESC cases using the following formula:

Risk score = (− 0.0423 × CD96) + (− 0.0040 × ITM2A) + (− 0.0446 × CLEC2D) + (0.0512 × SLC7A5) + (0.0051 × CCL5) + (− 0.0107 × FNBP1) + (0.2267 × TPM3) + (0.2069 × AMD1).

Then, based on the optimal cutoff value, CESC cases were divided into high TRS score group and low TRS score group (Supplementary Table S5-6). Gene set enrichment analysis (GSEA) revealed significant enrichment of several biological processes and pathways in the high-risk group. Notably, the KEGG pathways enriched include MAPK signaling, maturity onset diabetes of the young, melanoma, neuroactive ligand-receptor interaction, and ribosome (Fig. 6C; Supplementary Table S7).

**Fig. 7:**
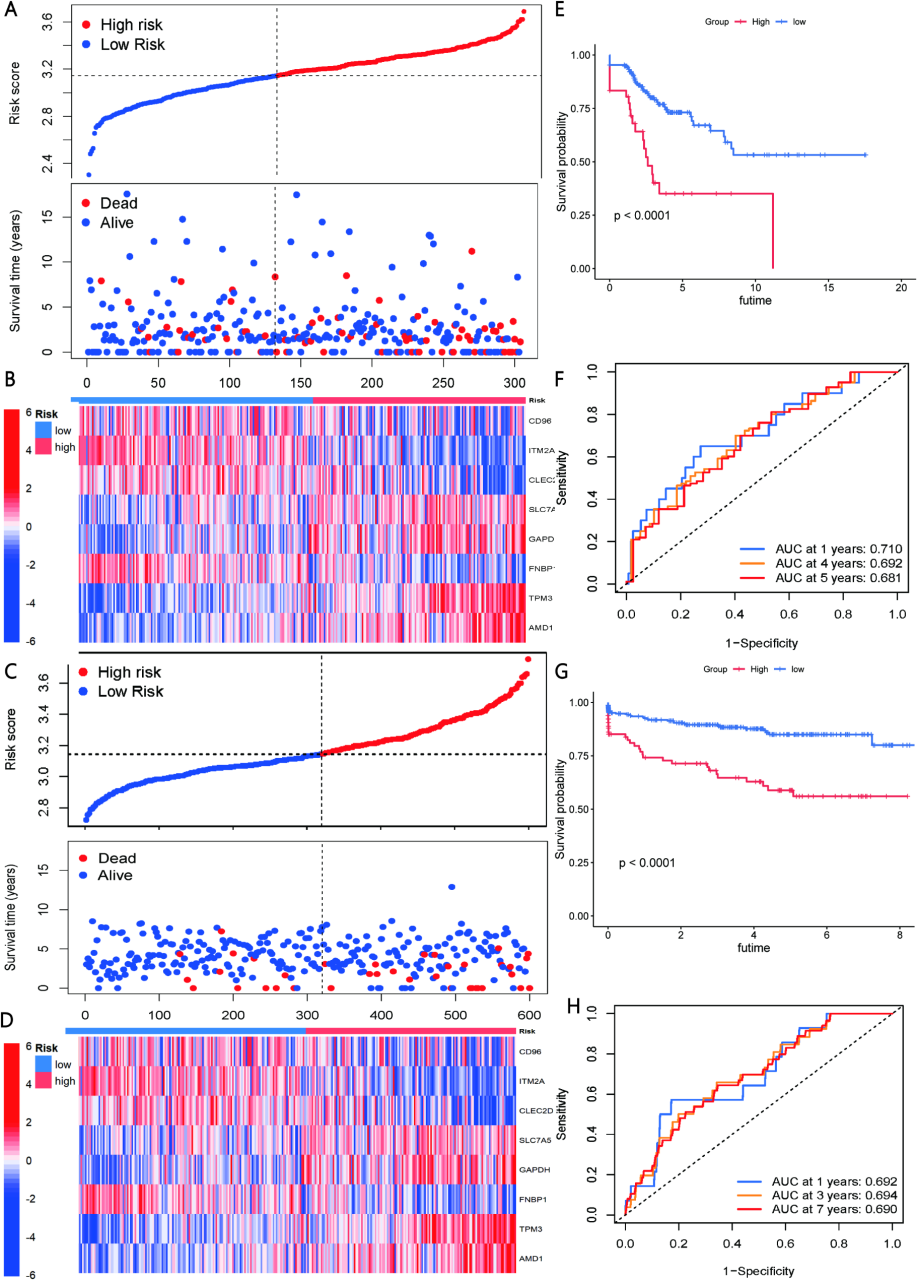
Validation of Prognostic Model. (**A**) Risk score distribution of high and low risk groups in the TCGA training cohort. (**B**) Expression patterns of prognostic genes in the TCGA training cohort. (**C**) Risk score distribution of high and low risk groups in the test cohort. (**D**) Expression patterns of prognostic genes in the test cohort. (**E**) K-M survival analysis of high and low risk groups in the TCGA training cohort. (**F**) ROC curve of the prediction model in the TCGA training cohort. (**G**) K-M survival analysis of high and low risk groups in the test cohort. (**H**) ROC curve of the prediction model in the test cohort

The ML-based prognostic risk scoring model we developed demonstrated robust predictive performance and clinical applicability in both the training and independent test cohorts of CESC patients. High-risk (red) and low-risk (blue) groups were identified in both cohorts, with high-risk scores correlating with shorter survival times (Fig. 7A and C). The prognostic genes exhibited significantly different expression patterns (Fig. 7B and D). Kaplan-Meier survival analysis confirmed the model’s prognostic predictive capability, indicating a significantly lower survival rate for patients in the high-risk group compared to the low-risk group (*p* < 0.0001) (Fig. 7E and G). Moreover, the model excelled in predicting the risk of death at various time points, with the AUCs in the training cohort for 1, 4, and 5 years ranging from 0.681 to 0.710 (Fig. 7F), and the AUCs in the test cohort for 1, 3, and 7 years being 0.692, 0.694, and 0.690, respectively (Fig. 7H).

### Correlation between TRS and immune cell infiltration and immune checkpoints

**Fig. 8:**
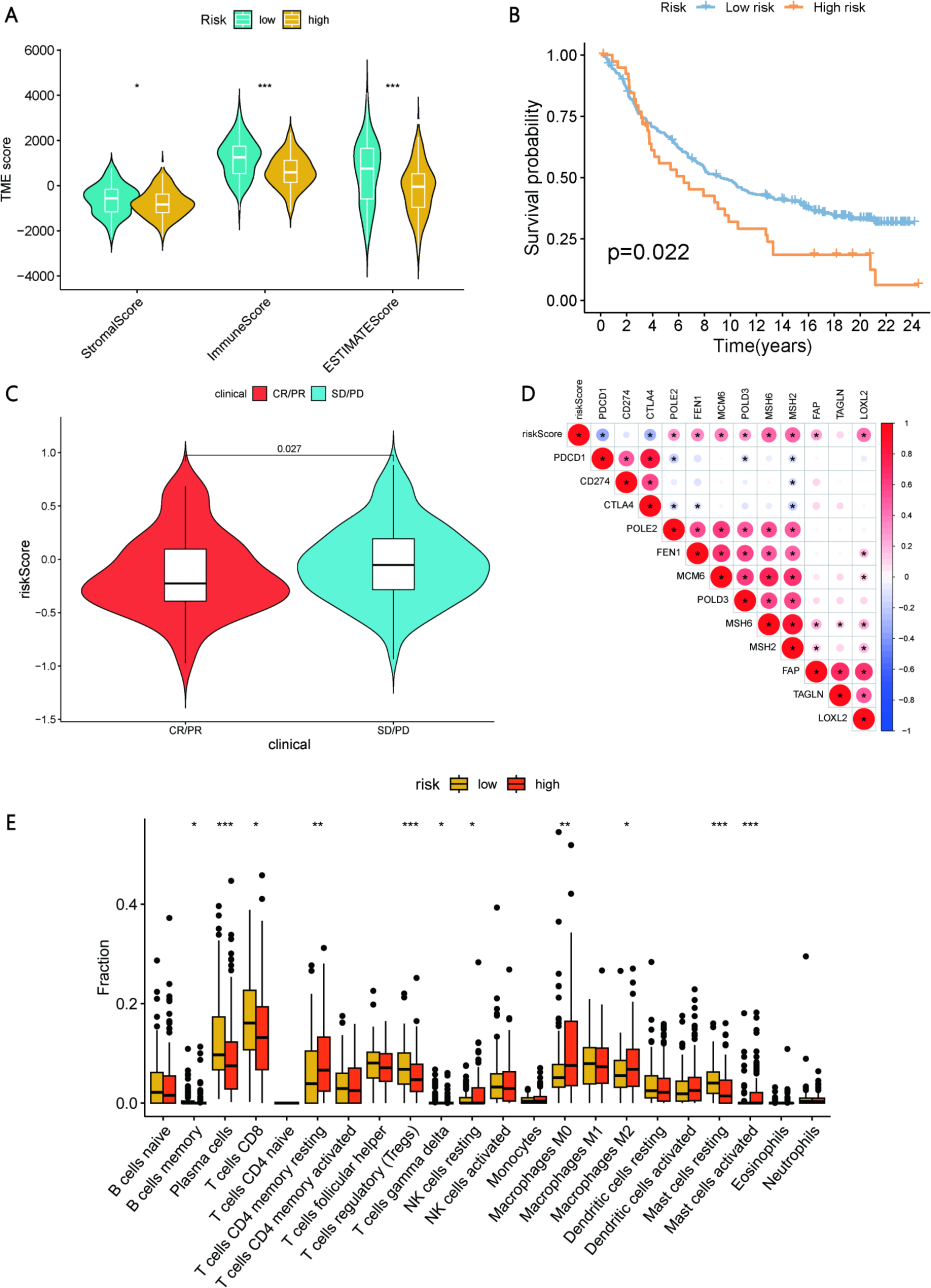
Comparison of Tumor Microenvironment Scores and Immune Cell Infiltration Levels Between High and Low-Risk Groups. (**A**) Comparison of tumor microenvironment scores between high and low-risk patients. (**B**) Kaplan-Meier survival curves of high and low-risk groups in the IMvigor210 cohort. (**C**) Risk score levels of patients with different treatment responses. (**D**)Correlation analysis between risk scores and related genes.(**E**) Immune cell infiltration levels in high and low-risk patients

We compared the TME scores and immune cell infiltration levels between the high-risk and low-risk groups. Figure 8A shows that the StromalScore, ImmuneScore, and ESTIMATEScore were significantly lower in high-risk patients compared to low-risk patients. In the IMvigor210 cohort, the Kaplan-Meier survival curve (Fig. 8B) indicates a significantly reduced survival rate for the high-risk group (*p* = 0.022). Figure 8C reveals that risk scores among different treatment response groups showed that patients in the CR (complete response) group had the lowest risk scores, while those in the SD (stable disease) and PD (progressive disease) groups had progressively higher risk scores. Correlation analysis (Fig. 8D) demonstrates that PDCD1 is significantly negatively correlated with the risk score, whereas POLE2 and FEN1 are significantly positively correlated. Furthermore, immune cell infiltration analysis indicates that the infiltration levels of various immune cells (such as CD8 + T cells, NK cells, plasma cells, and resting mast cells) were significantly lower in high-risk patients compared to low-risk patients (Fig. 8E; Supplementary Table S8).

### Virtual screening of drugs based on the drugbank database

To identify potential therapeutic drugs for CESC, we performed virtual screening and molecular docking analysis on 2,162 FDA-approved drugs from the DrugBank database. Eight core CD8T-related targets in CESC were selected for molecular docking. Based on the docking results, we focused on candidate drug molecules with binding affinities lower than − 6.9 (Supplementary Table S9). Considering docking scoring indicators such as binding affinity, RMSD/ub, RMSD/lb, along with the ranking and application of the drugs in cancer treatment, we ultimately selected sorafenib as a potential therapeutic candidate for CESC. In our molecular docking analysis, sorafenib exhibited strong binding ability with all eight core targets, with binding affinities ranging from − 6.9 to -9.0. Notably, it showed the strongest binding affinity with SLC7A5 -9.0. Additionally, sorafenib demonstrated stable binding conformations with multiple targets, indicated by low RMSD/ub and RMSD/lb values, suggesting a reliable binding mode.

### Visualization of Sorafenib binding modes with eight potential target proteins

**Fig. 9:**
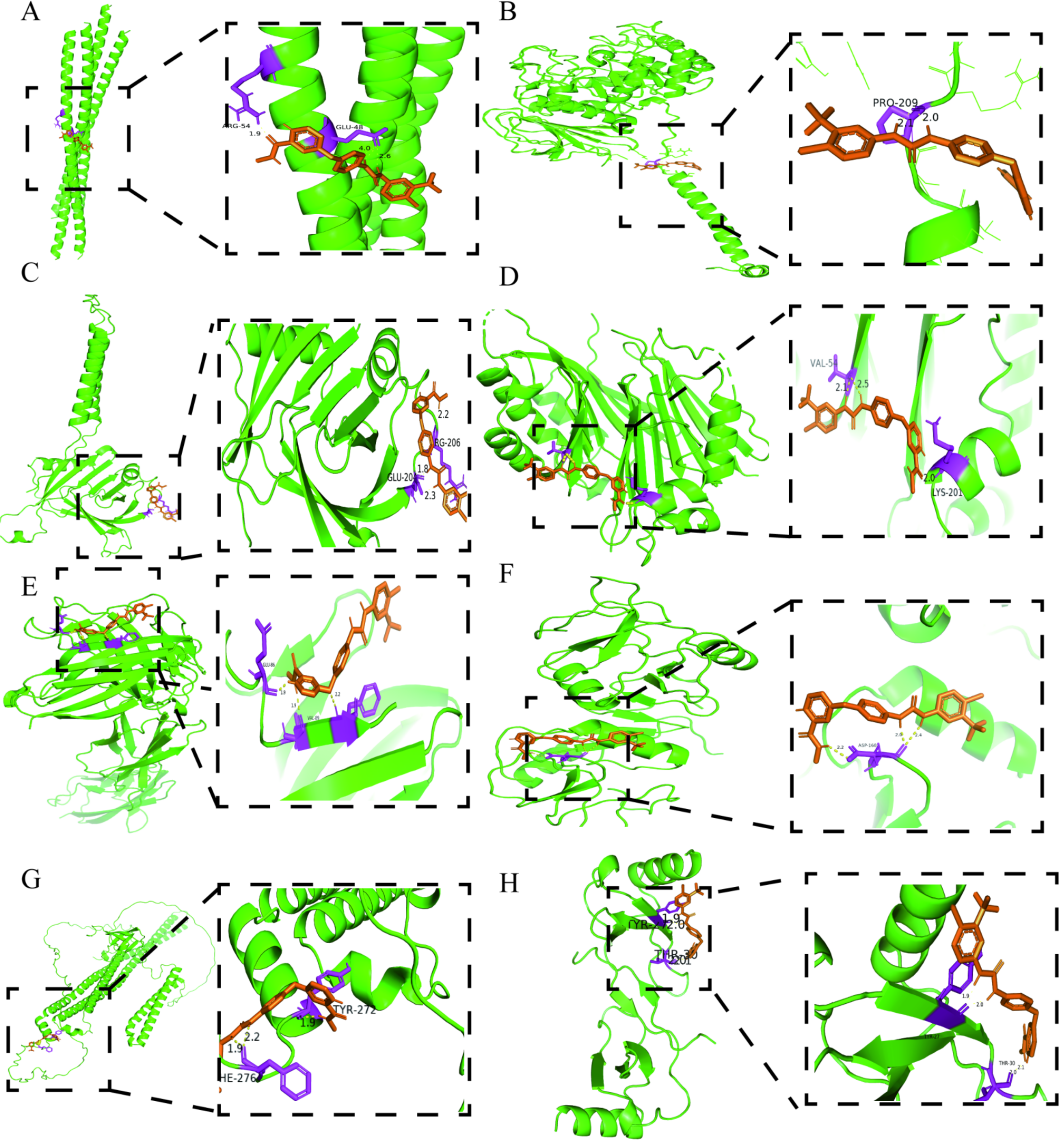
Molecular docking validation of sorafenib with target proteins. (**A**) TPM3; (**B**) SLC7A5; (**C**) ITM2A; (**D**) AMD1; (**E**) CD96; (**F**) CLEC2D; (**G**) FNBP1; (**H**) CCL5. Hydrogen bonds are indicated by yellow dashed lines. Sorafenib is shown in brown, the target proteins are shown in green, and the amino acid residues interacting through hydrogen bonds are highlighted in purple

We utilized PyMOL 2.5.4 to visualize the binding interactions between sorafenib and its eight potential target proteins (Fig. 9A–H). The results indicate that sorafenib primarily interacts with target proteins through hydrogen bond formation. Supplementary Table S10 summarizes the key interacting residues, hydrogen bond distances, and corresponding PDB IDs for each target protein. These interactions provide a fundamental basis for further elucidating the mechanism of action of sorafenib. The formation of hydrogen bonds may influence the conformation and function of target proteins, thereby modulating relevant signaling pathways and biological processes.

### Sorafenib enhances CD8 + T cell function and inhibits CESC cell invasion and viability

**Fig. 10:**
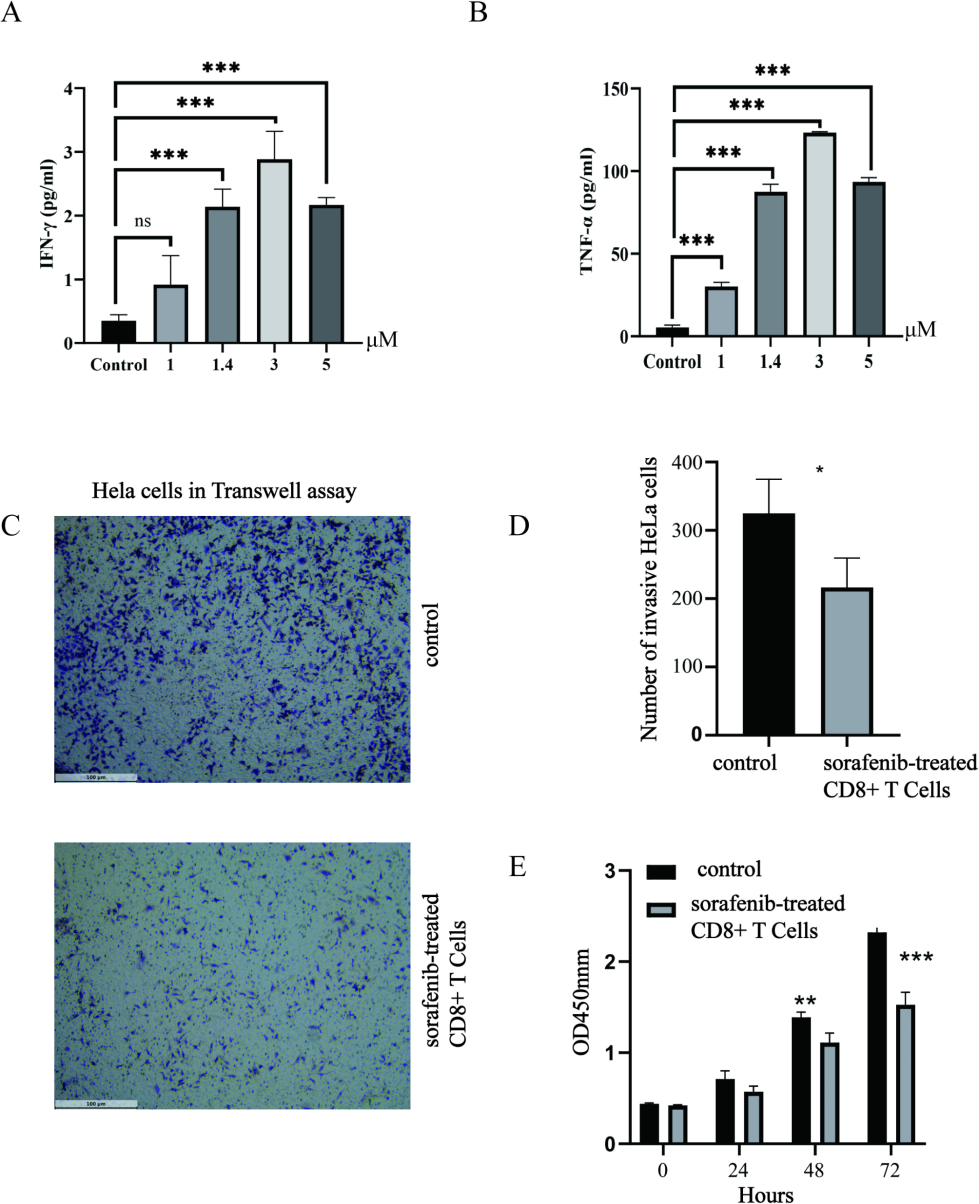
Sorafenib enhances the anti-tumor activity of CD8 + T cells. (**A**) The effect of different doses of sorafenib on IFN-γ secretion was assessed using ELISA. (**B**) The effect of different doses of sorafenib on TNF-α secretion was assessed using ELISA. (**C**) Representative images of invasive Hela cells in sorafenib-treated and control groups from the Transwell assay. (**D**) Quantitative analysis of the number of invasive Hela cells, comparing the sorafenib-treated group with the control group. (**E**) Co-culture experiments evaluating the inhibitory effects of sorafenib-treated CD8 + T cells on Hela cell viability

To select the most appropriate dose, we conducted experiments with varying concentrations, including 1 µM, 1.4 µM, 3 µM, and 5 µM, to ensure optimal immune response. ELISA analysis revealed that, compared to the control group, 3 µM sorafenib treatment significantly increased the secretion of key cytotoxic factors by CD8 + T cells. Figure 10A shows that treatment with 3 µM sorafenib significantly increased IFN-γ levels, indicating enhanced activation of CD8 + T cells. Similarly, Fig. 10B demonstrates a significant increase in TNF-α levels, which is directly associated with the cytotoxic potential of CD8 + T cells. Flow cytometry analysis revealed that, compared to the control group, the mean fluorescence intensity (MFI) of IFN-γ in CD8 + T cells from the sorafenib treatment group was significantly increased (*p* < 0.001), indicating that sorafenib enhances IFN-γ secretion by CD8 + T cells (Supplementary Figure S1A-B). Similarly, the MFI of TNF-α in CD8 + T cells from the sorafenib treatment group was also significantly higher than that in the control group (*p* < 0.001) (Supplementary Figure S1C-D). A Transwell invasion assay was used to evaluate the effect of sorafenib-treated CD8 + T cells on the invasive capacity of Hela cells. Figure 10C presents representative images of invasive Hela cells stained after 48 h, revealing a markedly reduced number of invasive cells in the sorafenib-treated CD8 + T cell group. Figure 10D further validated this result through quantitative analysis, showing that the number of invasive Hela cells in the sorafenib-treated group was significantly lower than that in the control group (*p* < 0.05). This suggests that sorafenib modulates the secretory activity of CD8 + T cells to influence the TME, thereby inhibiting CESC cell invasion. Co-culture experiments further assessed the cytotoxic effects of sorafenib-treated CD8 + T cells on Hela cells. Figure 10E shows that sorafenib treatment significantly reduced Hela cell viability in a time-dependent manner, particularly at 48 h (*p* < 0.01) and 72 h (*p* < 0.001).

In conclusion, our findings demonstrate that sorafenib enhances the secretion of IFN-γ and TNF-α by CD8 + T cells, thereby activating their cytotoxic function and inhibiting the invasion and survival of CESC cells. These results highlight the critical role of sorafenib in modulating the tumor immune microenvironment and exerting antitumor immunity, providing new experimental evidence and a theoretical basis for the combination of sorafenib with immunotherapy.

## Discussion

CESC is a major malignant tumor threatening women’s health [35]. Despite advances in treatment methods, patient prognosis remains poor. A comprehensive understanding of immune cell characteristics and functions, particularly CD8 + T cells, in the CESC microenvironment is crucial for developing novel immunotherapy strategies [3]. However, the current lack of systematic studies on CD8 + T cell heterogeneity and their role in the TME of CESC hinders the progress of precise immunotherapy [36]. This study innovatively integrates scRNA-seq and bulk RNA-seq data to systematically elucidate CD8 + T cell heterogeneity [37, 38], cell differentiation trajectories, regulatory networks, and intercellular communication in CESC. We identified key prognostic genes and constructed a CD8 + T cell-related prognostic risk model, demonstrating independent prognostic value. Our findings expand the understanding of CD8 + T cell mechanisms in CESC and provide new insights into the TME. This research highlights the significant potential of single-cell omics in precision oncology, aiming to advance the clinical application of precise immunotherapy.

The heterogeneity of CD8 + T cell subsets in CESC provides critical insights into their diverse roles within the TME. Our study identified four distinct CD8 + T cell subsets—CD8_termi, CD8_prolif, CD8_inter, and CD8_proge—each exhibiting unique marker expression and different functions in processes such as activation, proliferation, and differentiation. We found that the heterogeneity of CD8 + T cell subsets is closely associated with the clinical prognosis of CESC patients. The functional status of CD8 + T cells influences tumor immune evasion mechanisms and the response to immunotherapy. Consistent with existing literature, CD8 + T cells play a pivotal role in anti-tumor immunity. PD-1/PD-L1 immune checkpoint inhibitors, by relieving T cell suppression, restore and enhance the anti-tumor immune response of CD8 + T cells, making them a key strategy in CESC immunotherapy [39, 40]. Naive CD8 + T cells are activated upon encountering specific antigens, differentiate into highly cytotoxic effector cells to eliminate cancer cells, and partially transition into memory cells to ensure lasting immunity [41]. However, under chronic antigenic stimulation, such as within the TME, CD8 + T cells gradually become exhausted, impairing immune function. Huang’s findings highlight the critical roles of progenitor exhausted and tumor-specific memory CD8 + T cells, which cooperate during PD-1/PD-L1 checkpoint blockade therapy [42]. Additionally, KIR + CD8 + T cells act as a negative regulatory mechanism and are associated with poorer prognosis, whereas CD8_Ex_proge cells correlate with improved prognosis, and CD8_Ex_inter cells demonstrate strong cytotoxicity [34]. These observations underscore the complex and dynamic changes in CD8 + T cells, offering opportunities for precision immunotherapy. By enhancing effector functions while mitigating immune evasion, optimizing the balance of T cell subsets may ultimately improve patient outcomes.

This study reveals the specific communication patterns between CD8 + T cells at different differentiation stages and immune cells in the TME. It highlights key ligand-receptor pairs regulating the immune microenvironment, including CCL4-CCR5, CXCL9-DPP4, CTLA4-CD86, and CD28-CD80. These interactions demonstrate the balance of excitatory and inhibitory signals that regulate CD8 + T cell functions within the TME. Consistent with existing literature, GZMK + CD8 + T cells interact with CXCL12-secreting fibroblasts to promote neutrophil chemokine production, fueling inflammatory responses [43]. CXCL9 + tumor-associated macrophages enhance immune responses during early invasion stages through interactions with CD8 + Trm cells, while CXCL12 + tumor endothelial cells inhibit CD8 + T cell differentiation and attract myeloid-derived suppressor cells by secreting CXCL12, facilitating immune evasion [44]. Furthermore, the upregulation of CCR3/CCR5 in CD8 + T cells correlates with increased CCL5 secretion, suggesting a recruitment mechanism within the TME [45]. The regulation of CTLA4-CD86 signaling is critical for maintaining CD8 + T cell immune homeostasis [46]. These findings indicate that the interactions between CD8 + T cells and the TME play a pivotal role in balancing anti-tumor immunity and immune regulation, offering novel insights for optimizing immunotherapy strategies.

Transcription factors play a critical role in determining the anti-tumor immunity of CD8 + T cells by finely coordinating the expression of downstream target genes to regulate their activation, differentiation, proliferation, and effector functions in the TME. Our transcriptional network analysis revealed distinct regulatory patterns among CD8 + T cell subsets, with CD8_proge cells exhibiting enhanced TCF7, FOS, and RARA activity, while CD8_termi cells showed elevated ETV1, GATA3, and IRF4 activity. In comparison with existing literature, our study found that the FOS and c-Jun dimer influence CD8 + T cell activation and effector functions by regulating target gene expression. The AP-1 pathway regulated by c-Jun/FOS supports CD8 + T cell cytotoxicity and the persistence of immune memory during acute infection and anti-tumor immunity [47]. CD8 + cytotoxic T lymphocytes derived from central memory T cells highly express FOS, which is associated with proliferative capacity, anti-apoptotic properties, and sensitivity to IL-2/IL-15 signaling [48]. IRF4 regulates the proliferation of naive CD8 + T cells through AKT signaling, while the maintenance of memory cells is independent of IRF4. Elevated IRF4 promotes PD-1 expression and inhibits IFN-γ production, leading to an exhausted state in CD8 + T cells rather than simple activation [49]. Thus, regulation of these core factors provides mechanistic insights into the functional diversity of CD8 + T cells in tumor immunity and identifies potential targets for developing tumor therapies based on immune regulation.

CD8 + T cells, as key effector cells in the CESC microenvironment, play a crucial role in tumor development and prognosis. Previous studies have shown that the level of CD8 + T cell infiltration is an important prognostic marker for CESC. Higher levels of CD8 + T cell infiltration are generally associated with a better prognosis, while lower infiltration suggests a poorer prognosis [50]. In this study, survival analysis confirmed that a prognostic model based on CD8 + T cell-related genes can effectively distinguish the prognostic risk of CESC patients. The immune infiltration levels in high-risk patients were significantly lower than those in low-risk patients. This model demonstrated strong predictive performance in both the TCGA training cohort and the GSE57303 testing cohort, highlighting the importance of CD8 + T cell molecular characteristics in CESC prognosis assessment.

To identify potential candidate drugs for CESC immunotherapy, virtual screening and molecular docking analyses were conducted. The results confirmed that sorafenib stably binds with eight key CD8 + T cell-related targets, suggesting that sorafenib may modulate CD8 + T cell function through direct interaction with these critical molecules. Previous studies have supported its application in the immunotherapy of hepatocellular carcinoma and renal cell carcinoma, demonstrating strong anti-tumor efficacy and safety in clinical use [28, 29]. Based on these findings, we prioritized sorafenib as a candidate drug for CESC immunotherapy. The study indicates that sorafenib is a dual-target kinase inhibitor capable of simultaneously inhibiting the serine/threonine kinase Raf and the tyrosine kinases VEGFR/PDGFR, thereby exerting anti-tumor effects. Its mechanism of action involves two main aspects: first, by blocking the RAF/MEK/ERK signaling pathway, it directly inhibits tumor cell growth and proliferation; second, by suppressing VEGFR and PDGFR-mediated angiogenesis, it reduces tumor blood supply and indirectly inhibits tumor growth [51]. Furthermore, our research shows that sorafenib-treated CD8 + T cells significantly enhance the secretion of IFN-γ and TNF-α, thereby improving their cytotoxic activity. This result is consistent with studies in hepatocellular carcinoma, where elevated levels of IFN-γ⁺ CD8⁺ Ki67⁺ T cells after sorafenib treatment were closely associated with improved patient prognosis [28]. Mechanistically, sorafenib enhances the anti-tumor response of CD8 + T cells through dual pathways: on the one hand, it suppresses the activity of immunosuppressive cells by modulating the ERK MAPK and p38 MAPK signaling pathways; on the other hand, it promotes T cell infiltration into the TME [52]. Additionally, sorafenib inhibits STAT phosphorylation mediated by IFNα and IL-6, reducing PD-L1 expression and consequently diminishing tumor immune evasion. At the same time, sorafenib activates CD8 + T cells and enhances the expression of cytotoxic mediators GZMB and PRF1 [53]. These findings highlight the potential of sorafenib in the immunotherapy of cervical squamous cell carcinoma. As a potential drug for enhancing CD8 + T cell function, sorafenib could be used in combination with other immunotherapies to improve anti-tumor responses. Moreover, sorafenib demonstrates significant value in optimizing personalized treatment regimens, particularly in the TRS high-risk population identified in this study. These patients exhibit high expression of core targets, enabling sorafenib to effectively bind and regulate these targets, thereby achieving more pronounced therapeutic effects.

Although this study offers valuable insights into the heterogeneity of CD8 + T cells and their prognostic significance in the CESC microenvironment, several limitations must be acknowledged. First, all cohort studies included in this research are retrospective, necessitating further validation through prospective cohort studies. Second, the risk scoring model was developed using global single-cell RNA sequencing data, rather than CD8 + T cell-specific expression data obtained via deconvolution, which may dilute CD8 + T cell-specific signals and affect the model’s accuracy. Third, the limited number of single-cell RNA sequencing samples, combined with the available data from public databases, requires additional validation in larger, multi-center CESC cohorts. Lastly, the evaluation of sorafenib’s therapeutic efficacy is based primarily on molecular docking and in vitro experiments, without validation in animal models or clinical trials.

## Conclusion

This study integrates single-cell RNA sequencing and bulk RNA sequencing data, providing valuable insights into the heterogeneity and prognostic significance of CD8 + T cells in the CESC microenvironment. We identified distinct CD8 + T cell subpopulations and developed a predictive risk model based on CD8 + T cell-associated genes. The model demonstrates robust predictive performance and may aid in risk stratification and personalized treatment planning for CESC patients. Through virtual screening and molecular docking, we discovered that sorafenib might have immunotherapeutic potential in high-risk patients. This study enhances our understanding of the roles of CD8 + T cells in CESC and lays the foundation for developing precision immunotherapy strategies, highlighting the potential of multi-omics integration in advancing personalized cancer treatment.

## Electronic supplementary material

Below is the link to the electronic supplementary material.

Supplementary Material 1

Supplementary Material 2

Supplementary Material 3

Supplementary Material 4

Supplementary Material 5

Supplementary Material 6

Supplementary Material 7

Supplementary Material 8

Supplementary Material 9

Supplementary Material 10

Supplementary Material 11

## Data Availability

The following supporting information can be downloaded. Publicly available datasets were analyzed in this study. These include: scRNA-seq data (GSE171894) from GEO (www.ncbi.nlm.nih.gov/geo/), TCGA-CESC data (https://portal.gdc.cancer.gov), IMvigor210 cohort data (http://research-pub.gene.com/IMvigor210CoreBiologies), and the external validation dataset GSE44001 from GEO. Processed data are available upon reasonable request.

## References

[1] Bray F, Ferlay J, Soerjomataram I, Siegel RL, Torre LA, Jemal A. Global cancer statistics 2018: GLOBOCAN estimates of incidence and mortality worldwide for 36 cancers in 185 countries. CA Cancer J Clin. 2018;68(6):394–424. 30207593 10.3322/caac.21492

[2] Cohen PA, Jhingran A, Oaknin A, Denny L. Cervical cancer. Lancet. 2019;393(10167):169–82. 30638582 10.1016/S0140-6736(18)32470-X

[3] Binnewies M, Roberts EW, Kersten K, Chan V, Fearon DF, Merad M, Coussens LM, Gabrilovich DI, Ostrand-Rosenberg S, Hedrick CC, et al. Understanding the tumor immune microenvironment (TIME) for effective therapy. Nat Med. 2018;24(5):541–50. 29686425 10.1038/s41591-018-0014-x PMC5998822

[4] van der Leun AM, Thommen DS, Schumacher TN. CD8(+) T cell States in human cancer: insights from single-cell analysis. Nat Rev Cancer. 2020;20(4):218–32. 32024970 10.1038/s41568-019-0235-4 PMC7115982

[5] Tsukumo SI, Yasutomo K. Regulation of CD8(+) T cells and antitumor immunity by Notch signaling. Front Immunol. 2018;9:101. 29441071 10.3389/fimmu.2018.00101 PMC5797591

[6] Fukumura D, Kloepper J, Amoozgar Z, Duda DG, Jain RK. Enhancing cancer immunotherapy using antiangiogenics: opportunities and challenges. Nat Rev Clin Oncol. 2018;15(5):325–40. 29508855 10.1038/nrclinonc.2018.29 PMC5921900

[7] Azizi E, Carr AJ, Plitas G, Cornish AE, Konopacki C, Prabhakaran S, Nainys J, Wu K, Kiseliovas V, Setty M, et al. Single-Cell map of diverse immune phenotypes in the breast tumor microenvironment. Cell. 2018;174(5):1293–e13081236. 29961579 10.1016/j.cell.2018.05.060 PMC6348010

[8] Savas P, Virassamy B, Ye C, Salim A, Mintoff CP, Caramia F, Salgado R, Byrne DJ, Teo ZL, Dushyanthen S, et al. Single-cell profiling of breast cancer T cells reveals a tissue-resident memory subset associated with improved prognosis. Nat Med. 2018;24(7):986–93. 29942092 10.1038/s41591-018-0078-7

[9] Ostroumov D, Fekete-Drimusz N, Saborowski M, Kühnel F, Woller N. CD4 and CD8 T lymphocyte interplay in controlling tumor growth. Cell Mol Life Sci. 2018;75(4):689–713. 29032503 10.1007/s00018-017-2686-7 PMC5769828

[10] Gooden MJ, de Bock GH, Leffers N, Daemen T, Nijman HW. The prognostic influence of tumour-infiltrating lymphocytes in cancer: a systematic review with meta-analysis. Br J Cancer. 2011;105(1):93–103. 21629244 10.1038/bjc.2011.189 PMC3137407

[11] Wang Z, Gerstein M, Snyder M. RNA-Seq: a revolutionary tool for transcriptomics. Nat Rev Genet. 2009;10(1):57–63. 19015660 10.1038/nrg2484 PMC2949280

[12] Puram SV, Tirosh I, Parikh AS, Patel AP, Yizhak K, Gillespie S, Rodman C, Luo CL, Mroz EA, Emerick KS, et al. Single-Cell transcriptomic analysis of primary and metastatic tumor ecosystems in head and neck cancer. Cell. 2017;171(7):1611–e16241624. 29198524 10.1016/j.cell.2017.10.044 PMC5878932

[13] Wang H, Fu Y, Da BB, Xiong G. Single-Cell Sequencing Identifies the Heterogeneity of CD8 + T Cells and Novel Biomarker Genes in Hepatocellular Carcinoma. *J Healthc Eng* 2022, 2022:8256314. 10.1155/2022/8256314 PMC9018173 35449866

[14] Zhai Y, Zhang J, Huang Z, Shi R, Guo F, Zhang F, Chen M, Gao Y, Tao X, Jin Z, et al. Single-cell RNA sequencing integrated with bulk RNA sequencing analysis reveals diagnostic and prognostic signatures and Immunoinfiltration in gastric cancer. Comput Biol Med. 2023;163:107239. 37450965 10.1016/j.compbiomed.2023.107239

[15] Fridman WH, Pagès F, Sautès-Fridman C, Galon J. The immune contexture in human tumours: impact on clinical outcome. Nat Rev Cancer. 2012;12(4):298–306. 22419253 10.1038/nrc3245

[16] Baudino TA. Targeted cancer therapy: the next generation of cancer treatment. Curr Drug Discov Technol. 2015;12(1):3–20. 26033233 10.2174/1570163812666150602144310

[17] Wilhelm S, Carter C, Lynch M, Lowinger T, Dumas J, Smith RA, Schwartz B, Simantov R, Kelley S. Discovery and development of Sorafenib: a multikinase inhibitor for treating cancer. Nat Rev Drug Discov. 2006;5(10):835–44. 17016424 10.1038/nrd2130

[18] Yau T, Park JW, Finn RS, Cheng AL, Mathurin P, Edeline J, Kudo M, Harding JJ, Merle P, Rosmorduc O, et al. Nivolumab versus Sorafenib in advanced hepatocellular carcinoma (CheckMate 459): a randomised, multicentre, open-label, phase 3 trial. Lancet Oncol. 2022;23(1):77–90. 34914889 10.1016/S1470-2045(21)00604-5

[19] Butler A, Hoffman P, Smibert P, Papalexi E, Satija R. Integrating single-cell transcriptomic data across different conditions, technologies, and species. Nat Biotechnol. 2018;36(5):411–20. 29608179 10.1038/nbt.4096 PMC6700744

[20] Zhang J, Lu T, Lu S, Ma S, Han D, Zhang K, Xu C, Liu S, Gan L, Wu X, et al. Single-cell analysis of multiple cancer types reveals differences in endothelial cells between tumors and normal tissues. Comput Struct Biotechnol J. 2023;21:665–76. 36659929 10.1016/j.csbj.2022.12.049 PMC9826920

[21] Trapnell C, Cacchiarelli D, Grimsby J, Pokharel P, Li S, Morse M, Lennon NJ, Livak KJ, Mikkelsen TS, Rinn JL. The dynamics and regulators of cell fate decisions are revealed by pseudotemporal ordering of single cells. Nat Biotechnol. 2014;32(4):381–6. 24658644 10.1038/nbt.2859 PMC4122333

[22] Van de Sande B, Flerin C, Davie K, De Waegeneer M, Hulselmans G, Aibar S, Seurinck R, Saelens W, Cannoodt R, Rouchon Q, et al. A scalable SCENIC workflow for single-cell gene regulatory network analysis. Nat Protoc. 2020;15(7):2247–76. 32561888 10.1038/s41596-020-0336-2

[23] Liu Z, Liu L, Weng S, Guo C, Dang Q, Xu H, Wang L, Lu T, Zhang Y, Sun Z, et al. Machine learning-based integration develops an immune-derived LncRNA signature for improving outcomes in colorectal cancer. Nat Commun. 2022;13(1):816. 35145098 10.1038/s41467-022-28421-6 PMC8831564

[24] Newman AM, Liu CL, Green MR, Gentles AJ, Feng W, Xu Y, Hoang CD, Diehn M, Alizadeh AA. Robust enumeration of cell subsets from tissue expression profiles. Nat Methods. 2015;12(5):453–7. 25822800 10.1038/nmeth.3337 PMC4739640

[25] Mariathasan S, Turley SJ, Nickles D, Castiglioni A, Yuen K, Wang Y, Kadel EE III, Koeppen H, Astarita JL, Cubas R, et al. TGFβ attenuates tumour response to PD-L1 Blockade by contributing to exclusion of T cells. Nature. 2018;554(7693):544–8. 29443960 10.1038/nature25501 PMC6028240

[26] Subramanian A, Tamayo P, Mootha VK, Mukherjee S, Ebert BL, Gillette MA, Paulovich A, Pomeroy SL, Golub TR, Lander ES, et al. Gene set enrichment analysis: a knowledge-based approach for interpreting genome-wide expression profiles. Proc Natl Acad Sci U S A. 2005;102(43):15545–50. 16199517 10.1073/pnas.0506580102 PMC1239896

[27] Guedes IA, de Magalhães CS, Dardenne LE. Receptor-ligand molecular Docking. Biophys Rev. 2014;6(1):75–87. 28509958 10.1007/s12551-013-0130-2 PMC5425711

[28] Kalathil SG, Hutson A, Barbi J, Iyer R, Thanavala Y. Augmentation of IFN-γ + CD8 + T cell responses correlates with survival of HCC patients on Sorafenib therapy. JCI Insight 2019, 4(15). 10.1172/jci.insight.130116 PMC6693832 31391334

[29] Yao J, Xi W, Chen X, Xiong Y, Zhu Y, Wang H, Hu X, Guo J. Mast cell density in metastatic renal cell carcinoma: association with prognosis and tumour-infiltrating lymphocytes. Scand J Immunol. 2021;93(4):e13006. 33275792 10.1111/sji.13006

[30] Ling K, Dou Y, Yang N, Deng L, Wang Y, Li Y, Yang L, Chen C, Jiang L, Deng Q, et al. Genome editing mRNA nanotherapies inhibit cervical cancer progression and regulate the immunosuppressive microenvironment for adoptive T-cell therapy. J Control Release. 2023;360:496–513. 37423524 10.1016/j.jconrel.2023.07.007

[31] Wang Y, Wang Z, Jia F, Xu Q, Shu Z, Deng J, Li A, Yu M, Yu Z. CXCR4-guided liposomes regulating hypoxic and immunosuppressive microenvironment for sorafenib-resistant tumor treatment. Bioact Mater. 2022;17:147–61. 35386453 10.1016/j.bioactmat.2022.01.003 PMC8965090

[32] Iyer RV, Maguire O, Kim M, Curtin LI, Sexton S, Fisher DT, Schihl SA, Fetterly G, Menne S, Minderman H. Dose-Dependent sorafenib-Induced immunosuppression is associated with aberrant NFAT activation and expression of PD-1 in T cells. Cancers (Basel) 2019, 11(5). 10.3390/cancers11050681 PMC6562672 31100868

[33] Jin K, Zhao D, Zhou J, Zhang X, Wang Y, Wu Z. Pulsed electromagnetic fields inhibit IL-37 to alleviate CD8(+) T cell dysfunction and suppress cervical cancer progression. Apoptosis. 2024;29(11–12):2108–27. 39404933 10.1007/s10495-024-02006-8

[34] Sui S, Tian Y, Wang X, Zeng C, Luo OJ, Li Y. Single-cell RNA sequencing gene signatures for classifying and scoring exhausted CD8(+) T cells in B-cell acute lymphoblastic leukaemia. Cell Prolif. 2024;57(3):e13583. 38030593 10.1111/cpr.13583 PMC10905324

[35] Arbyn M, Weiderpass E, Bruni L, de Sanjosé S, Saraiya M, Ferlay J, Bray F. Estimates of incidence and mortality of cervical cancer in 2018: a worldwide analysis. Lancet Glob Health. 2020;8(2):e191–203. 31812369 10.1016/S2214-109X(19)30482-6 PMC7025157

[36] Chung HC, Ros W, Delord JP, Perets R, Italiano A, Shapira-Frommer R, Manzuk L, Piha-Paul SA, Xu L, Zeigenfuss S, et al. Efficacy and safety of pembrolizumab in previously treated advanced cervical cancer: results from the phase II KEYNOTE-158 study. J Clin Oncol. 2019;37(17):1470–8. 30943124 10.1200/JCO.18.01265

[37] Mimitou EP, Cheng A, Montalbano A, Hao S, Stoeckius M, Legut M, Roush T, Herrera A, Papalexi E, Ouyang Z, et al. Multiplexed detection of proteins, transcriptomes, clonotypes and CRISPR perturbations in single cells. Nat Methods. 2019;16(5):409–12. 31011186 10.1038/s41592-019-0392-0 PMC6557128

[38] Zhang Z, Wang ZX, Chen YX, Wu HX, Yin L, Zhao Q, Luo HY, Zeng ZL, Qiu MZ, Xu RH. Integrated analysis of single-cell and bulk RNA sequencing data reveals a pan-cancer stemness signature predicting immunotherapy response. Genome Med. 2022;14(1):45. 35488273 10.1186/s13073-022-01050-w PMC9052621

[39] Meng Y, Liang H, Hu J, Liu S, Hao X, Wong MSK, Li X, Hu L. PD-L1 expression correlates with tumor infiltrating lymphocytes and response to neoadjuvant chemotherapy in cervical cancer. J Cancer. 2018;9(16):2938–45. 30123362 10.7150/jca.22532 PMC6096364

[40] Heeren AM, Rotman J, Stam AGM, Pocorni N, Gassama AA, Samuels S, Bleeker MCG, Mom CH, Zijlmans H, Kenter GG, et al. Efficacy of PD-1 Blockade in cervical cancer is related to a CD8(+)FoxP3(+)CD25(+) T-cell subset with operational effector functions despite high immune checkpoint levels. J Immunother Cancer. 2019;7(1):43. 30755279 10.1186/s40425-019-0526-z PMC6373123

[41] Koh CH, Lee S, Kwak M, Kim BS, Chung Y. CD8 T-cell subsets: heterogeneity, functions, and therapeutic potential. Exp Mol Med. 2023;55(11):2287–99. 37907738 10.1038/s12276-023-01105-x PMC10689838

[42] Huang Q, Xu L, Ye L. Functional subsets of tumor-specific CD8(+) T cells in draining lymph nodes and tumor microenvironment. Curr Opin Immunol. 2024;92:102506. 39591663 10.1016/j.coi.2024.102506

[43] Guo CL, Wang CS, Wang ZC, Liu FF, Liu L, Yang Y, Li X, Guo B, Lu RY, Liao B, et al. Granzyme K(+)CD8(+) T cells interact with fibroblasts to promote neutrophilic inflammation in nasal polyps. Nat Commun. 2024;15(1):10413. 39614076 10.1038/s41467-024-54685-1 PMC11607458

[44] Ren YF, Ma Q, Zeng X, Huang CX, Ren JL, Li F, Tong JJ, He JW, Zhong Y, Tan SY, et al. Single-cell RNA sequencing reveals immune microenvironment niche transitions during the invasive and metastatic processes of ground-glass nodules and part-solid nodules in lung adenocarcinoma. Mol Cancer. 2024;23(1):263. 39580469 10.1186/s12943-024-02177-7 PMC11585206

[45] Smyth LJ, Starkey C, Gordon FS, Vestbo J, Singh D. CD8 chemokine receptors in chronic obstructive pulmonary disease. Clin Exp Immunol. 2008;154(1):56–63. 18727632 10.1111/j.1365-2249.2008.03729.x PMC2561084

[46] Lang TJ, Nguyen P, Peach R, Gause WC, Via CS. In vivo CD86 Blockade inhibits CD4 + T cell activation, whereas CD80 Blockade potentiates CD8 + T cell activation and CTL effector function. J Immunol. 2002;168(8):3786–92. 11937530 10.4049/jimmunol.168.8.3786

[47] Papavassiliou AG, Musti AM. The multifaceted output of c-Jun biological activity: focus at the junction of CD8 T cell activation and exhaustion. Cells 2020, 9(11). 10.3390/cells9112470 PMC7697663 33202877

[48] Wang X, Wong CW, Urak R, Taus E, Aguilar B, Chang WC, Mardiros A, Budde LE, Brown CE, Berger C, et al. Comparison of Naïve and central memory derived CD8(+) effector cell engraftment fitness and function following adoptive transfer. Oncoimmunology. 2016;5(1):e1072671. 26942092 10.1080/2162402X.2015.1072671 PMC4760301

[49] Hirsch T, Neyens D, Duhamel C, Bayard A, Vanhaver C, Luyckx M, Sala de Oyanguren F, Wildmann C, Dauguet N, Squifflet JL, et al. IRF4 impedes human CD8 T cell function and promotes cell proliferation and PD-1 expression. Cell Rep. 2024;43(7):114401. 38943641 10.1016/j.celrep.2024.114401

[50] Enwere EK, Kornaga EN, Dean M, Koulis TA, Phan T, Kalantarian M, Köbel M, Ghatage P, Magliocco AM, Lees-Miller SP, et al. Expression of PD-L1 and presence of CD8-positive T cells in pre-treatment specimens of locally advanced cervical cancer. Mod Pathol. 2017;30(4):577–86. 28059093 10.1038/modpathol.2016.221

[51] Deng Q, Huang Y, Zeng J, Li X, Zheng X, Guo L, Shi J, Bai L. Recent advancements in the small-molecule drugs for hepatocellular carcinoma (HCC): Structure-activity relationships, Pharmacological activities, and the clinical trials. Biomed Pharmacother. 2024;179:117343. 39180795 10.1016/j.biopha.2024.117343

[52] Sunay MM, Foote JB, Leatherman JM, Edwards JP, Armstrong TD, Nirschl CJ, Hicks J, Emens LA. Sorafenib combined with HER-2 targeted vaccination can promote effective T cell immunity in vivo. Int Immunopharmacol. 2017;46:112–23. 28282575 10.1016/j.intimp.2017.02.028 PMC5811992

[53] Cheng CC, Ho AS, Peng CL, Chang J, Sie ZL, Wang CL, Chen YL, Chen CY. Sorafenib suppresses radioresistance and synergizes radiotherapy-mediated CD8(+) T cell activation to eradicate hepatocellular carcinoma. Int Immunopharmacol. 2022;112:109110. 36037651 10.1016/j.intimp.2022.109110

