# Supplementary material for: Single-cell transcriptomic analysis reveals CD8 + T cell heterogeneity and identifies a prognostic signature in cervical cancer
Source: BMC Cancer. 2025 Mar 18;25:498. doi: 10.1186/s12885-025-13901-x (PMC11916872; doi:10.1186/s12885-025-13901-x)
Supplement: Supplementary file 1 — Supplementary Material 1 [file 12885_2025_13901_MOESM1_ESM.pdf]

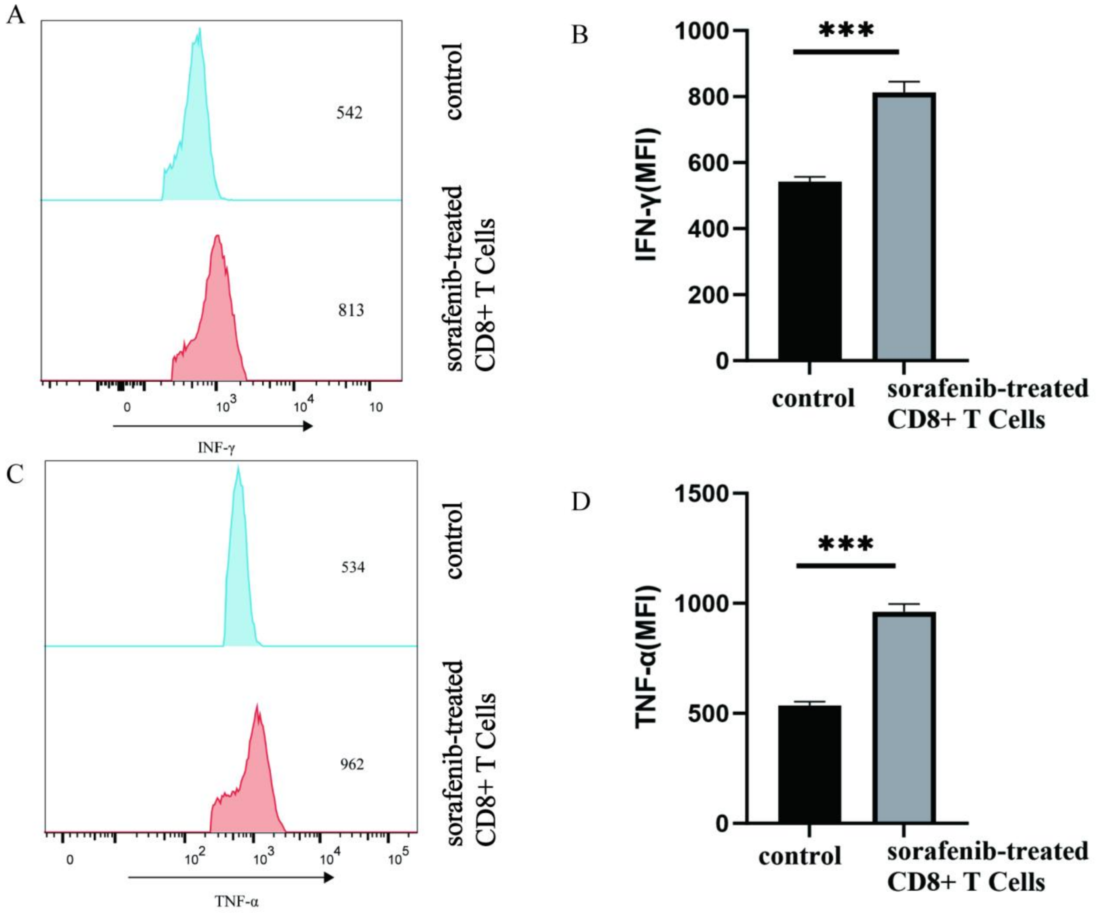

Supplementary Figure S2. Sorafenib Enhances the Pro-inflammatory Function of CD8+ T Cells. (A, B) The mean fluorescence intensity of IFN-γ production in CD8+ T cells from the control and sorafenib-treated groups. (C, D) The mean fluorescence intensity of TNF-α production in CD8+ T cells from the control and sorafenib-treated groups.
