# Supplementary material for: Single-cell transcriptomic analysis reveals CD8 + T cell heterogeneity and identifies a prognostic signature in cervical cancer
Source: BMC Cancer. 2025 Mar 18;25:498. doi: 10.1186/s12885-025-13901-x (PMC11916872; doi:10.1186/s12885-025-13901-x)
Supplement: Supplementary file 11 — Supplementary Material 11 [file 12885_2025_13901_MOESM11_ESM.pdf]

	Core Target	residue	distance of hydrogen bonds	PDB ID
Sorafenib	TPM3	ARG-54	1.9Å	6otn
			4.0Å	
		GLU-48	2.6Å	
	SLC7A5	PRO-209	2.0Å	6irs
			2.1Å	
	ITM2A	GLU-20	1.8Å	AF-043736-F1
			2.3Å	
		ARG-206	2.2Å	
	AMD1	LYS-201	2.0Å	1i72
		VAL-54	2.1Å	
			2.5Å	
	CD96	GLU-86	1.9Å	6arq
			2.9Å	
		VAL-89	2.4Å	
	CLEC2D	ASP-168	2.4Å	4qki
			2.0Å	
			2.2Å	
	FNBP1	TYR-272	1.9Å	2efl
			2.2Å	
		PHE-276	1.9Å	
	CCL5	TYR-27	2.1Å	1b3a
			1.8Å	
		THR-30	2.3Å	

Supplementary Table S10. Key interactions between Sorafenib and potential target proteins.
